# Breaking scaling relationships in alkynol semi-hydrogenation by manipulating interstitial atoms in Pd with *d*-electron gain

**DOI:** 10.1038/s41467-022-30540-z

**Published:** 2022-05-18

**Authors:** Yang Yang, Xiaojuan Zhu, Lili Wang, Junyu Lang, Guohua Yao, Tian Qin, Zhouhong Ren, Liwei Chen, Xi Liu, Wei Li, Ying Wan

**Affiliations:** 1 The Education Ministry Key Laboratory of Resource Chemistry, Joint International Research Laboratory of Resource Chemistry of Ministry of Education, Shanghai Key Laboratory of Rare Earth Functional Materials, and Shanghai Frontiers Science Center of Biomimetic Catalysis, Shanghai Normal University, Shanghai, 200234 China; 2 School of Physical Science and Technology, Shanghai Tech University, Shanghai, 201210 China; 3 School of Chemistry and Chemical Engineering, In-situ Center for Physical Sciences, Shanghai Jiao Tong University, Shanghai, 200240 China; 4 School of Chemistry and Chemical Engineering, Frontiers Science Center for Transformative Molecules, Shanghai Jiao Tong University, Shanghai, 200240 China; 5 Department of Chemistry, Laboratory of Advanced Materials, Shanghai Key Laboratory of Molecular Catalysis and Innovative Materials, iChEM and State Key Laboratory of Molecular Engineering of Polymers, Fudan University, Shanghai, 200433 China

**Keywords:** Heterogeneous catalysis, Catalyst synthesis, Chemical engineering

## Abstract

Pd catalysts are widely used in alkynol semi-hydrogenation. However, due to the existence of scaling relationships of adsorption energies between the key adsorbed species, the increase in conversion is frequently accompanied by side reactions, thereby reducing the selectivity to alkenols. We report that the simultaneous increase in alkenol selectivity and alkynol conversion is achieved by manipulating interstitial atoms including B, P, C, S and N in Pd catalysts. A negative linear relationship is observed between the activation entropies of 2-methyl-3-butyn-2-ol and 2-methyl-3-buten-2-ol which is highly related to the filling of *d*-orbital of Pd catalysts by the modification of *p*-block elements. A catalyst co-modified by B and C atoms has the maximum *d* charge of Pd that achieves a 17-fold increase in the turn-over frequency values compared to the Lindlar catalysts in the semi-hydrogenation of 2-methyl-3-butyn-2-ol. When the conversion is close to 100%, the selectivity can be as high as 95%.

## Abstract (web summary)

Circumventing the linear scaling relationship in the semi-hydrogenation is challenging. Here the authors report a method for breaking the scaling relationships using ordered mesoporous carbon-supported Pd nanocatalysts with *d*-electron gain by *p*-block atoms occupying interstitial sites in the lattice as a greener alternative to Lindlar catalysts for the selective hydrogenation of alkynols.

## Introduction

A palladium nanocatalyst supported on solid is a more preferred hydrogenation catalyst in the industry than homogeneous catalysts in terms of green chemistry and reusability of the catalysts, mainly taking advantage of the adsorption and dissociation of hydrogen molecules (H_2_) to form adsorbed H atoms (H_ad_) over Pd surface^1^. Semi-hydrogenation of alkynols to alkenols is one of the most widely studied industrially applicable catalytic processes in producing vitamins, medicines, fragrances, artificial fibers, etc^2^. One of the steps in the synthesis of unsaturated alcohols, for example, isophytol for the further production of vitamin E, is the selective hydrogenation of 2-methyl-3-butyn-2-ol (MBY) to 2-methyl-3-buten-2-ol (MBE)^3^. In addition, MBE can be used in the preparation of vitamin A, citronellol, ionones, farnesol, and sesquiterpenes^4^. However, the formation of an undesired over-hydrogenation product (alkanol) is simultaneously promoted by Pd, particularly at high conversions^5,6^.

Modifiers are often used to increase the selectivity in the semi-hydrogenation, including the incorporation of additives to the Pd nanoparticles which induce variations in the Pd electronic (e.g., charge transfer) and/or geometric (e.g., separation of Pd surface sites) properties^7,8^. For example, Lindlar catalysts in which Pd is modified with lead acetate and quinoline have been developed and are widely used in the semi-hydrogenation of acetylene to ethylene. On one hand, the modification with quinoline may influence the polarization of the Pd-H bond in the proximity of the catalyst surface due to possible electron donation^9^. On the other hand, the poisoning effect or separation of Pd surface sites by Pb may result in a decrease of the adsorption of acetylene and ethylene. This effect increases ethylene selectivity but lowers the conversion. In addition, modification of the surface with other metals such as Ag, Au, and Cu and also subsurface carbon leads to weaker adsorption of CH_3_ groups and hence other carbon species, including acetylene and ethylene^10^. This phenomenon is related to the energy linear scaling relationships (LSRs) that are linear correlations between different surface bond energies for the adsorbed species^11,12^. Significant attention has been paid to break such relationships in both theoretical and experimental aspects using modified catalysts due to both electronic and geometric effects. The latter at the atomistic level could be considered as related to the former. In most cases, the changes mostly affect the viability of the reaction network^13^. For example, the adsorption mode of OOH intermediates in the direct hydrogenation of O_2_ to H_2_O_2_ can be rearranged from planar configuration to vertical configuration by electrostatic interactions and hydrogen bonds^14^. The lack of vertically adsorbed bonds to the surface prevents effective dissociation and excellent selectivity and conversion can be achieved simultaneously.

Modifying Pd metal with *p*-block elements has been used to break LSRs in selective hydrogenations, but the number of studies is very limited. The catalysts include Pd_x_S/B prepared by a post modification of supported metal nanoparticles with Na_2_S^15^ or borane tetrahydrofuran (BH_3_·THF)^16^, Pd with subsurface B prepared by inducing B from the porous boron nitride carrier to metal^17^, etc. (Supplementary Fig. 1). In addition, Pd can also be modified with subsurface C during the hydrogenation of acetylene^18^. General optimization of the metal nanocatalysts remains a significant challenge, partially because of the lack of understanding of the properties of complex electronic structure materials. The easy recycling of Pd nanocatalysts in the industry by combustion of carbon support has stimulated our study of the carbon-supported interstitial Pd catalysts that contain easily removal of light element atoms. A versatile synthesis approach for industrially usable Pd nanocatalysts, and more importantly, a quantitative correlation of surface electronic structure to conversion and selectivity for transition metal interstitial catalysts by a simple and experimentally measurable descriptor are greatly needed to break the linear relationships.

Here, we explore a reverse atom diffusion approach to synthesize Pd interstitial nanocatalysts with a series of *p*-block atoms including B, P, C, S, and N as sustainable alternatives to Lindlar catalysts for selective hydrogenation. The well-defined structure allows for a direct determination of the structure-property relationship on the basis of the *d*-electron gain of Pd catalysts by the modification of *p*-block elements. An increase in the *d* charge of Pd induces the rearrangement of MBE from an adsorption configuration through C=C bonds to one using the hydroxyl group (-OH) terminations which reduces the activation entropy. This phenomenon, together with the enhanced adsorption entropy of MBY, breaks the scaling relationships, and favors a simultaneous improvement of both the conversion and selectivity of MBY semi-hydrogenation. The finding is a very important advance in rational catalyst design based on electronic descriptors.

## Results

### Structure of Pd interstitial nanocatalysts with *p*-block atoms

A reverse atom diffusion approach combined with triblock copolymer self-assembly is used to synthesize Pd nanocatalysts with interstitial atoms including B, P, C, S, and N. This approach uses low-polymerized phenolic resin as a carbonaceous source, triblock copolymer F127 as a structure-directing agent, triphenyl borate (triphenyl phosphite, citric acid, mercaptosuccinic acid, urea) as a doping and coordination agent, and palladium chloride as a metal source. All the chemicals used are commercial. The polymerization of resins and their subsequent carbonization under the protection of nitrogen has been realized in the industry. Therefore, this strategy is easily reproducible and can be scaled up, providing a greener alternative to Lindlar catalysts which use toxic Pb(OAc)_2_ and suffer the leaching of surface-doped element Pb in the presence of products^19^.

**Fig. 1:**
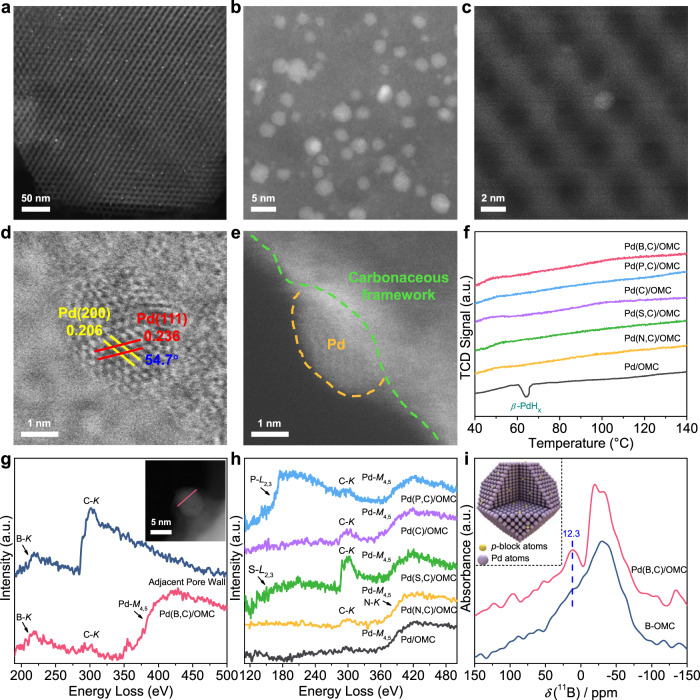
Structure of Pd nanocatalysts. **a**–**c** Representative AC-ADF-STEM images, **d** AC-ABF-STEM image, **e** high-resolution SE-STEM image in STEM mode for Pd(B,C)/OMC. **f** TPHD curves for Pd interstitial nanocatalysts with different interstitial atoms and Pd/OMC reference catalyst without interstitial atoms. **g** EELS spectra for Pd(B,C)/OMC. For reference, the EELS spectrum for adjacent carbonaceous pore wall is also provided. Inset in **g** is the line scanning for a selected Pd nanoparticle. **h** EELS spectra for interstitial Pd nanocatalysts containing P/C, C, S/C, and N/C. For reference, the EELS spectra for pure Pd/OMC are also provided. **i** ^11^B solid-state NMR spectra of Pd(B,C)/OMC (red line) and B-OMC without Pd (navy line). Inset is the schematic of Pd nanoparticles modified with *p*-block atoms.

Transmission electron microscope (TEM) images for all Pd interstitial nanocatalysts have hexagonally arranged elongated nanopores with well-distributed nanoparticles (Supplementary Fig. 2a–e). Aberration-corrected angle annular dark-field scanning transmission electron-microscopy (AC-ADF-STEM) images also show uniform nanoparticles in large domains and no metal single atoms were observed (Fig. 1a–c). The particle sizes were measured to be approximately 5.0 nm, in good agreement with the pulsing CO chemisorption results (Supplementary Table 1). Interestingly, semi-exposed Pd nanoparticles are observed from STEM images in both annular bright-field (ABF) and secondary electron (SE) modes, partially embedded in the walls of a carbonaceous pore and partially exposed to the pore channel (Fig. 1d, e). This feature suggests a moderate interaction between carbon and Pd and also the availability of active sites to the reactants. Wide-angle X-ray diffraction (XRD) patterns show a broad diffraction peak at ~39°, which is attributed to the very small Pd nanoparticles (Supplementary Fig. 3). A distinct shift of this peak to a lower angle for all Pd nanocatalysts with non-metal atoms indicates lattice expansion of Pd due to the presence of non-metal interstitial atoms^20^. It has been reported that a subsurface *β*-PdH_X_ phase is easily formed in supported Pd nanocatalysts with H situated in the subsurface octahedral sites of the Pd^18^. As expected, a reference Pd/OMC catalyst which was synthesized by the impregnation and has 4.7 nm Pd nanoparticles dispersing in ordered mesopores (Supplementary Fig. 2f), shows the evolution of H_2_ at ~60 °C due to the decomposition of *β*-PdH_X_ in the temperature-programmed hydride decomposition (TPHD) curves (Fig. 1f), confirming the presence of subsurface H^21^. However, this peak disappears in the patterns for all the Pd nanocatalysts with non-metal interstitial atoms, suggesting the substitution of the subsurface H by non-metal interstitial atoms^22^.

In order to determine the elemental composition of Pd nanoparticles in Pd(B,C)/OMC, an individual particle with no covering layers was analyzed by electron energy loss spectroscopy (EELS) (Fig. 1g). The B-*K* and C-*K* edges associated with the Pd-*M*_4,5_ edge can be clearly detected, which suggests that B and C are taken up by the Pd nanoparticle. Similarly, it is proved that other Pd interstitial nanocatalysts contain dopant atoms of P/C, C, and S/C (Fig. 1h). However, the determination of interstitial N is difficult due to the overlap of N-*K* and Pd-*M*_4,5_ edges. In contrast, no obvious non-metal-*K* edge is observed in Pd/OMC, suggesting pure Pd nanoparticles with no non-metal atoms. The presence of interstitial atoms is also proved by solid-state nuclear magnetic resonance (NMR) spectra. The ^11^B solid-state NMR data of Pd(B,C)/OMC show an additional resonance centered at 12.3 ppm compared to B-doped ordered mesoporous carbon (B-OMC), which is attributed to the interstitial B species in Pd lattice (Fig. 1i)^23^. The final catalysts contain similar Pd and dopant amounts as shown by XPS and EDX results (Supplementary Table 1).

### Electronic properties

**Fig. 2:**
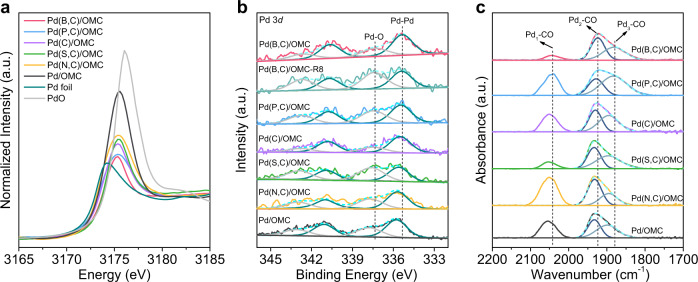
Electronic properties. **a** XANES spectra of the Pd *L*_3_-edge of Pd interstitial nanocatalysts, reference Pd/OMC catalyst, and reference samples (Pd foil and PdO). **b** XPS spectra of the 3*d* level of Pd with Pd binding energies fitting by peak fitting programs. Pd(B,C)/OMC-R8 is the Pd(B,C)/OMC catalyst after eight catalytic runs. **c** CO-DRIFTS spectra with schemes identifying the distinct adsorption modes for Pd interstitial nanocatalysts and the reference Pd/OMC catalyst.

The Pd *L*_3_-edge X-ray absorption near edge structure (XANES) spectra of interstitial Pd catalysts and reference post-impregnated Pd nanocatalyst are shown in Fig. 2a together with those of a Pd foil and PdO. The absorption edge clearly shifts to higher energies for the post-impregnated Pd nanocatalyst compared to that of a Pd foil. In addition, the white line peak is more intense and shifts to higher energy. Similar results have been observed for the Pt clusters, and these effects are attributable to the development of a band gap and localization of empty state wavefunctions in small clusters rather than oxidized samples^24^. As a result, the electronic properties of the interstitial nanocatalysts are estimated from the XANES difference between them and the reference post-synthesized Pd catalyst with similar particle sizes to exclude the size effect. An obvious reduced white line intensity is observed for the interstitial catalysts, while the edge energy almost remains unchanged. Since the white line intensity depends on both the density of the unoccupied states, as well as the transition probabilities to them from the 2*p* orbital, the reduced intensity may be related to the orbital hybridization that decreases the density of empty states. Therefore, this phenomenon is assigned to the formation of interstitial atoms in the Pd lattice, the hybridization of orbitals, and the depletion of empty *d* states. In addition, the white line can be quantitatively understood in terms of transitions to the unoccupied high density of states of the *d*-band^25,26^. The change of *d* accounts is calculated, ranging from 0 to 0.48 e, which is connected to the distribution of the unoccupied states of *d* states and represents a major proportion of all the 4*d* characters above the Fermi level. The X-ray photoelectron spectroscopy (XPS) spectra of the interstitial Pd show that the 3*d* core levels shift to lower binding energy compared to the pure Pd catalyst (Fig. 2b). A shift of 0.4 eV is observed for Pd(B,C) relative to that of pure Pd nanoparticles, which is attributed to interstitial atom-Pd interaction^27,28^, which may include a “charge transfer” between atoms in the character of core-level electrons of surface atoms through *sp*-*d* hybridization.

CO adsorption at 298 K over the reference Pd/OMC resulted in adsorption maxima at 2055, 1932, and 1899 cm^−1^ in the CO diffuse reflectance infrared Fourier transform spectra (CO-DRIFTS), which are assigned to linearly bound CO, bridged CO (Pd_2_-CO) and CO molecules bound to three palladium atoms (Pd_3_-CO), respectively^29^ (Fig. 2c). A redshift of the stretching wavenumber of adsorbed CO, which is sensitive to the electron density of transition surfaces, is observed for Pd interstitial nanocatalysts. Similar results have been observed on the Pd surface modified by organic groups or Ag. Electron donation from the organic groups or Ag to Pd leads to an increase in the number of 4*d* electrons feeding back to the π^*^ antibonding orbital in CO, resulting in a weaker C=O bond^30,31^. Considering the similar particle sizes of the interstitial catalysts and the reference, we also contribute the redshift of the CO adsorption band to an increase in the number of Pd *d* electrons, in good agreement with the XANES results. The CO adsorption is reduced over B-doped Pd compared to unmodified Pd, similar to the results for B-doped Pd supported on porous boron nitride, but with a weaker extent compared to the latter^17^. This is possibly originated from the concentrations of B. The synthesis of B-doped Pd with controllable subsurface B concentrations in Pd and ensemble structures to optimize the *d* charge is deserved further investigation.

### Catalytic performance for semi-Hydrogenation of alkynols over Pd nanocatalysts

**Fig. 3:**
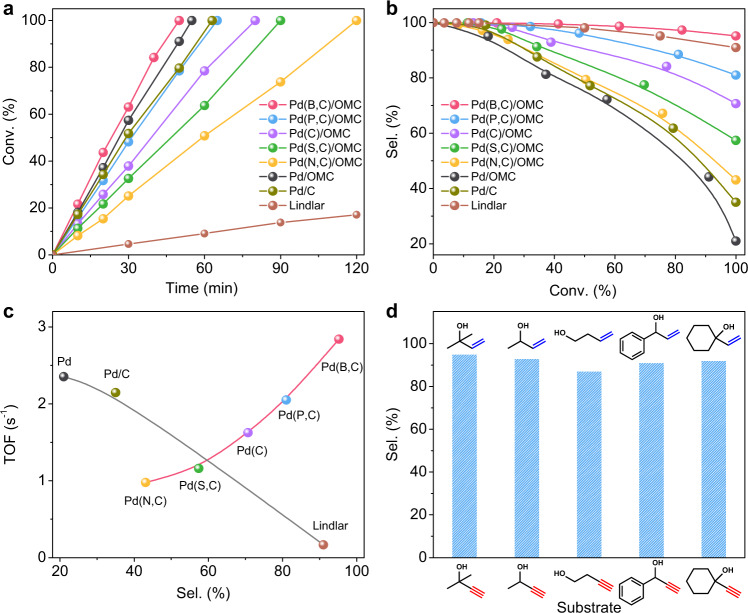
Kinetics. **a** MBY conversion (Conv.) as a function of time, **b** compile of the selectivity (Sel.) to MBE as a function of MBY conversion, and **c** relationship between the TOF values and selectivity of MBE on the studied Pd nanocatalysts. **d** Semi-hydrogenation of various alkynols on Pd(B,C)/OMC. The reaction conditions were: 0.045 mol% Pd; 1.25 mmol of the substrate; 5 mL of ethanol; 25 °C; 800 rpm; and in the presence of hydrogen by an H_2_ balloon under atmospheric pressure.

As shown in Fig. 3a, b, the interstitial catalyst co-modified with B and C atoms (Pd(B,C)/OMC) shows excellent conversion and selectivity in the semi-hydrogenation of MBY to MBE at 298 K. The turn-over frequency (TOF) value reach 2.84 s^−1^, which is 17 times that of a commercial Lindlar catalyst (0.17 s^−1^), and selectivity of ~95% to MBE was achieved at a conversion of ~100%, slightly higher than Lindlar catalyst (91%). In contrast, the post-impregnated Pd nanocatalyst in the absence of interstitial atoms shows a high TOF value (14 times that of a Lindlar catalyst), but a much low selectivity (21%). It is obvious that the LSR is broken, achieving both a high TOF value and selectivity over Pd(B,C)/OMC, compared to Lindlar catalysts and a post-impregnated Pd nanocatalyst (Fig. 3c). When the interstitial atom is P, C, S, and N, the catalysts show a moderate TOF value and selectivity and interestingly, a higher TOF value corresponding to a higher selectivity. For example, the TOF value is 2.05 s^−1^ for Pd(P,C)/OMC, and the selectivity is 81% at a conversion of ~100%, both are slightly lower than Pd(B,C)/OMC. Pd(N,C)/OMC shows a reduced TOF value of 0.98 s^−1^; and a selectivity of 43% but still two times higher than the pure post-impregnated Pd catalyst. The only detectable by-product at a higher conversion for all catalysts is 2-methyl-3-butan-2-ol (MBA).

To confirm the heterogenous catalysis, the leaching of Pd was first investigated. Solid trapping tests using mesoporous silica SH-SBA-15, containing mercapto functional groups as the trapping agent, were performed to exclude the effect of leached Pd (Supplementary Fig. 4). Once soluble palladium is released from the catalyst into the solution and serves as active species, mercapto functional groups could easily and efficiently capture it, and therefore quench the reactions^32,33^. No significant difference in the TOF value over Pd nanocatalysts regardless of the type of interstitial atom was observed in the presence of SH-SBA-15 compared to the reaction without it, confirming negligible Pd leaching into the solution for interstitial Pd nanocatalysts. Second, hot filtration experiments were carried out (Supplementary Fig. 5). The catalysts were hot filtered during the reaction and the conversion remained constant. Third, the reusability of Pd(B,C)/OMC was tested. The initial reaction rate (*r*_0_) which is dominated by the overall number of active sites, the overall conversion, and the selectivity to MBE at a high conversion remained almost unchanged in 8 successive runs, confirming no change in the active Pd concentrations (Supplementary Fig. 6). Fourth, the reused Pd(B,C)/OMC catalyst was characterized. The Pd concentrations were determined by inductively coupled plasma-atomic emission spectrometry (ICP-AES), the particle size characterized by CO chemisorption and TEM, and the electronic structure analysed by XPS, were analogous to those of the fresh catalyst (Fig. 2b and Supplementary Table 1), indicating the stability of the Pd nanoparticles with no leaching, aggregation, and poisoning. As a result, the heterogenous reaction occurring on the Pd surface is the best model for semi-hydrogenation.

The diffusion limitation was first excluded to study the kinetics. The influence of the stirring rate on MBY hydrogenation rate was investigated to exclude external diffusion limitations. The degree of MBY conversion was independent of the stirring rate, indicating no mass transfer limitations above 700 rpm on our system (Supplementary Fig. 7a). In addition, Pd(B,C)/OMC were synthesized with similar particle sizes (5 nm) but different Pd loadings (Supplementary Figs. 8 and 9). The reaction rate of MBY increased linearly with Pd concentrations ranging from 44 to 105 μmol g_cat_^−1^ (Supplementary Fig. 7b), which indicates the exclusion of internal diffusion and the achievement of the kinetic regime for the mesoporous catalyst due to the ordered, large mesopore channels and high surface areas (Supplementary Fig. 10 and Supplementary Table 1). The linear conversion plot as shown in Fig. 3a indicates a zero-order reaction for MBY. A constant *r*_0_ in the range 0.10–0.55 mol L^−1^ MBY under constant H_2_ pressure and temperature, confirms a zero-order for MBY in the rate equation (Supplementary Fig. 11a). The change in initial hydrogen pressures while keeping the MBY concentration constant shows that the overall reaction is 1/2-order for H_2_ (Supplementary Fig. 11b). The kinetic isotopic effect (KIE) was tested to investigate the rate-determining step. The experimentally observed *k*_H_/*k*_D_ ratio (the rate constant (*k*) with H_2_ or D_2_) is ~1.2 for all interstitial catalysts (Supplementary Fig. 12), which suggests that H_2_ dissociation is not the rate-determining step^34^, which is also in agreement with most hydrogenation reactions involved with the homolytic dissociation of H_2_^35^.

The reaction rates for MBY (*k*_1_) and MBE (*k*_2_) are compared for Pd nanocatalysts assuming that the reaction involves two-step hydrogenation as reported in the literature for semi-hydrogenation, and the Arrhenius plots for each step are shown in Supplementary Fig. 13. The apparent activation energies (*E*_a_) and entropies (Δ*S*^0*^) are estimated based on the Arrhenius equation and transition state theory (Supplementary information). A linear relationship was observed for *E*_a_ and Δ*S*^0*^ for Pd catalysts modified by non-metal elements for both the selective hydrogenation of MBY to MBE and of MBE to MBA (Supplementary Fig. 14). This compensation effect may be due to the freedom of the system increases with the energy of the system increasing^36–38^. The energies associated with the bonding of the surface intermediate can therefore be estimated from the entropy change^39,40^. The increase in the binding energy of MBY for a modified Pd surface with the order of Pd(B,C) > Pd(P,C) > Pd(C) > Pd(S,C) > Pd(N,C), is reflected by the increase of Δ*S*^0*^ value which leads to a greater restriction of the freedom of the C≡C bond in MBY. The hydrogenation is accelerated. In contrast, the binding of the C=C bond in MBE for further hydrogenation follows the reverse order, reducing the hydrogenation rate.

The general scope for the selective hydrogenation of various functionalized alkynols was then extended. It was interesting to find that Pd(B,C)/OMC served as a high-efficiency catalyst with high selectivity to alkenols production (Fig. 3d).

### DFT calculations

The effect of the chemical modifications produced by the interstitial atoms on the electronic structure of palladium was confirmed by density functional theory (DFT) calculations. When boron is found on the surface of Pd(111), it can diffuse into the subsurface (Supplementary Fig. 15a), occupying the subsurface sites (octahedral sites) rather than surface sites (*hcp* sites). In fact, boron atoms are known to be most stable in octahedral interstitial sites of palladium^41^. Similar results have been found for interstitial P and C atoms (Supplementary Fig. 15b, c), the subsurface octahedral sites being much favored on Pd(111) which is consistent with previously reported results^42^. In contrast, penetration into surface *hcp* sites may more favorable for N and S, possibly due to the large difference in electronegativity (Supplementary Fig. 15d, e). As a result, the more favorable configuration is an *hcp* site on the S-Pd(111) and N-Pd(111) surfaces. The energy relative to the Fermi level was also calculated (Supplementary Fig. 16). The bonding states formed by the hybridization of *s*, *p*, and *d*-bands of Pd(111) are located below the Fermi level, and no clear hybridization peaks were observed above the Fermi level. After interstitial atoms dissolve into the metal lattice, an effective overlap between the *d*-band of Pd atoms and the *p*-band of interstitial atoms (B, P, C, S, and N) appears. The energy of the *d*-band of the Pd atom is broader and positioned below the Fermi level. At the same time, there is a new bonding state for interstitial Pd positioned at 5–7 eV below the Fermi level, showing that the localized bonding between non-metal interstitial atoms and Pd induces the filling of the Pd *d*-band, thus shifting it away from the Fermi edge^29,43^.

**Fig. 4:**
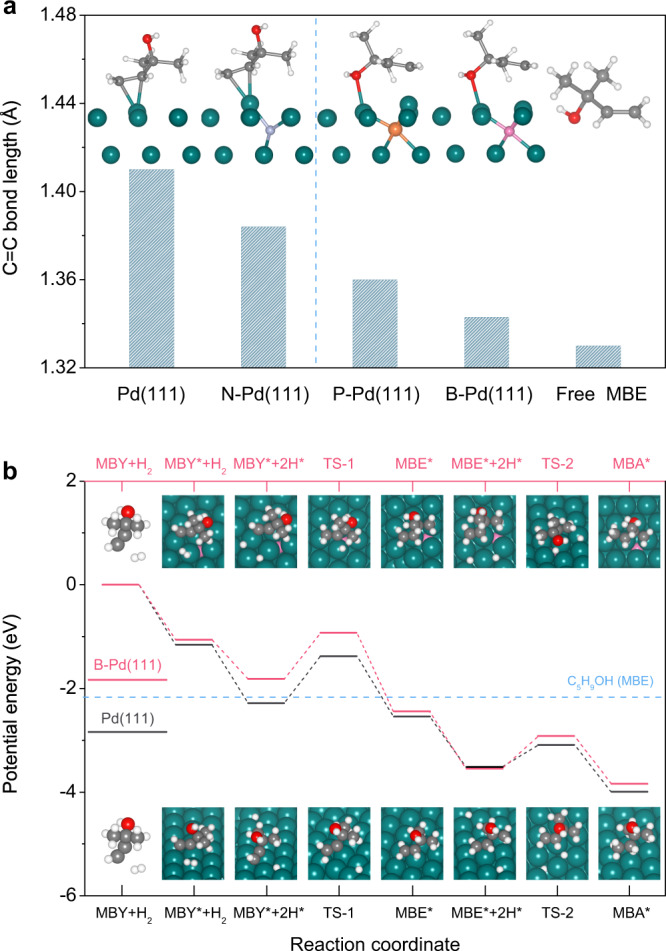
DFT calculations. **a** The most favorite adsorption configuration and C=C bond length in MBE on the pure Pd(111), and interstitial atoms (B, P, and N) modified Pd(111) surfaces (Pd cyan, B pink, P orange, N blue, C gray, O red, H white). **b** Reaction pathway for the semi-hydrogenation of MBY on the pure Pd(111) and B-Pd(111) surfaces. The geometry structure of involved intermediates and transition states (TS) are illustrated.

The adsorption configurations for the reactant MBY and the product MBE on interstitial atom-modified Pd(111) surfaces are shown in Fig. 4a and Supplementary Tables. 2, 3, respectively. On Pd(111), the adsorption configurations show that MBY tends to bond with surface Pd atoms through a di-*σ* configuration, while MBE is inclined to bond in a *π*-bonded configuration through a C=C bond, while -OH tilts away from the surface. The C≡C and C=C bond lengths are calculated to be 1.33 and 1.41 Å, respectively, much longer than in free molecules. When there are many subsurface non-metal atoms, there is a high degree of similarity to what is found on a pure Pd(111) surface for MBY with changes in the adsorption energy. In addition, the MBE configuration over N-modified Pd(111) is similar to that over pristine Pd(111). However, the configuration changes for the adsorption of MBE in the presence of interstitial B and P atoms and adopts a configuration using the -OH, and the C=C bond length is estimated to be about 1.34 Å, very close to that in a free MBE molecule. The change in configurations indicates a significant change in the electronic structure of surface Pd atoms after modifying with B and P atoms.

To provide insight into the hydrogenation pathway, the energy profiles for the hydrogenation of MBY are given (Fig. 4b). Two-step hydrogenation is suggested with an intermediate of MBE over the pure Pd(111). The activation barrier for the first hydrogenation from MBY to MBE is calculated to be 0.91 eV. B-modified Pd(111) shows a similar barrier, indicating that these two surfaces have similar hydrogenation activities, analogous to the experiment results. The difference occurs at the next hydrogenation step in the presence of B. The hydrogenation of MBE can proceed over Pd(111) due to the lack of an obvious difference in the desorption and hydrogenation barriers (0.37 vs. 0.42 eV). In contrast, MBE prefers desorption rather than to be hydrogenated (0.27 vs. 0.63 eV) over B-Pd(111) which is possibly related to the adsorption configuration with an unactivated C=C bond.

It should be noted that diffusion of dissociated H into the subsurface to form *β*-PdH_X_ is observed in TS-2 on Pd(111). This species is active in hydrogenation. In contrast, H cannot diffuse into the subsurface due to the presence of interstitial B. As a result, the activation barrier for further hydrogenation is increased in the latter.

## Discussion

A reverse atom diffusion approach was used to synthesize Pd nanocatalysts with interstitial atoms including B, P, C, S, and N. The possible path of interstitial atoms doping was verified by thermogravimetric analysis-mass spectrometry (TG-MS) analysis (Supplementary Fig. 17). The molecules released during thermal reduction contain BH_3_CO, PH_3_, C_2_H_2_, H_2_S, and NH_3_, respectively. These gaseous molecules can adsorb on the surface of Pd and deposit the corresponding atoms (B, P, C, S, and N), which gradually dissolve and diffuse into the Pd lattice so that a dynamic equilibrium is established^18,44–46^. The interstitial atoms occupy the surface *hcp* sites and subsurface octahedral sites. It is challenging to experimentally determine the relative amounts of dopant incorporated for each element. We assume that this value may not greatly differ from each other due to the similar adsorption energies of different atoms on the Pd surface, the high-temperature carbothermal reduction for atom diffusion, and the same occupation of the surface/subsurface sites in the Pd lattice.

These interstitial catalysts show an interesting positive relationship between semi-hydrogenation of alkynol TOF value and alkenol selectivity, which is in the general negative for Pd nanocatalysts due to the LSR. For example, the acetylene and ethylene adsorption energies have been found to scale with the carbon-surface bond energy as measured by the adsorption energy of CH_3_ groups^10^. The best catalyst will be a compromise between selectivity and TOF value. In the present case, this linear scaling relationship is broken by alternative energy forms which is significantly affected by the electronic structure of metal catalyst^13^.

**Fig. 5:**
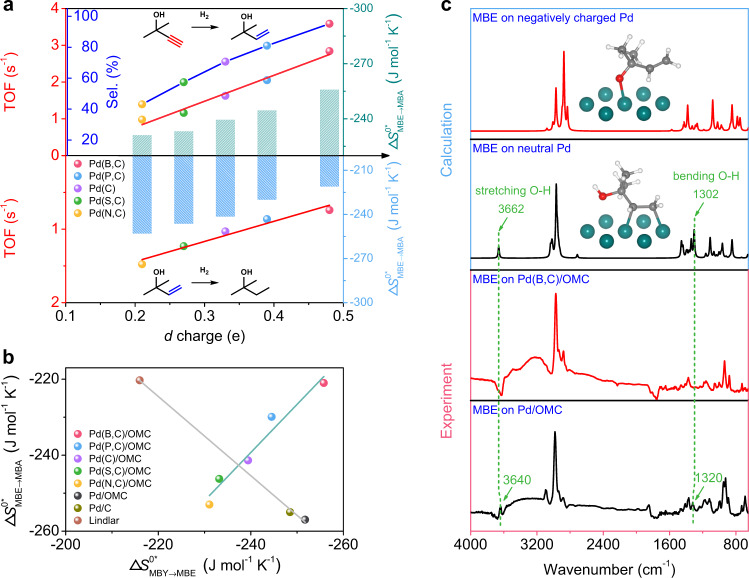
Breaking the scaling relationships. **a** Relationship between the *d* charge of Pd sites and TOF (red line)/MBE selectivity (blue line) of the Pd interstitial nanocatalysts, and the relationship between the *d* charge of Pd sites and the Δ*S*^0*^ for the semi-hydrogenation of MBY to MBE (light cyan column) or MBE to MBA (light blue column). **b** Relationship between the Δ*S*^0*^ for the semi-hydrogenation of MBY to MBE and MBE to MBA. **c** In situ FT-IR spectra for MBE on Pd(B,C)/OMC and Pd/OMC and DFT calculations for MBE interaction with neutral and negatively charged Pd atoms (set 4/7 extra electron per Pd). In (**c**), the cyan, red, gray, and white balls represent Pd, O, C, and H atoms, respectively.

The modification of the electronic structure would vary as the *d*-band is modified by non-metal atoms that transfer *s* or *p* electrons into the *d*-band of Pd. The adsorption configuration and energy are largely related to the electronic structure^47–50^. Here, in particular, the *d* charge, which reflects the filling of *d*-band holes of Pd and has been calculated earlier, is used to explore the relationships between electronic structure and adsorption and in turn catalytic properties. The activation entropy associated with the surface adsorption and reaction was then considered which has been reported to be related to the availability of electronic energy levels in the solid^51,52^. Figure 5a shows plots of activation entropies of MBY and MBE hydrogenation vs*. d* charge of Pd. Thus, Δ*S*^0*^ for the chemoselective hydrogenation of MBY to MBE increases smoothly with an increase in the number of electrons which occupy the *d*-levels. This can be seen to directly influence the strength of C≡C bonds to Pd, which becomes stronger as the *d*-occupancy increases. The TOF value for MBY hydrogenation increases linearly. In contrast, the adsorption of the C=C functional group becomes weaker as the *d* charge increases. The TOF value for MBE hydrogenation is dramatically reduced. A negative linear relationship was found between Δ*S*^0*^_MBY→MBE_ for the chemoselective hydrogenation of MBY to MBE and Δ*S*^0*^_MBE→MBA_ for MBE to MBA (Fig. 5b). That is, a higher Δ*S*^0*^_MBY→MBE_ corresponds to a lower Δ*S*^0*^_MBE→MBA_. These results are in good agreement with the DFT calculations that the adsorption configuration transforms from the di-*σ* adsorption using the C=C bonds to perpendicular adsorption using the O-H bonds over B-modified Pd(111), and the desorption for MBE is energetically favorable compared to the further hydrogenation by C=C bonds. Easy dissociation of reactants and weak binding of intermediates are key factors for efficient and selective hydrogenation. In contrast, the Lindlar catalysts, the commercial Pd/C catalysts, and the post-impregnated Pd/OMC catalyst with no interstitial atoms follow a reverse linear relationship, as similarly reported in literatures^53,54^. Therefore, an alternative adsorption configuration on Pd nanoparticles due to an increase of *d* electron gain breaks the LSR.

In situ Fourier transform infrared spectroscopy (FT-IR) spectra (Fig. 5c) were also performed to identify the adsorption configuration on the interstitial Pd nanocatalyst. Two bands at 3640 and 1320 cm^−1^ are observed for Pd/OMC from the stretching and the bending vibrations of the -OH, respectively, suggesting the -OH tilts away from the surface. However, these two bands disappear for Pd(B,C)/OMC, indicating that the activation of -OH and dissociative adsorption occurs. DFT calculations were then carried out to simulate the charge effect of Pd on the adsorption of MBE. When the Pd atom is neutral, MBE strongly interacts with Pd through the broadened *π* orbitals of C=C bonds which makes a major contribution to the highest occupied molecular orbital (HOMO) of MBE (Supplementary Fig. 18), rather than through the hydroxyl group. In contrast, the negatively charged Pd preferentially reacts with -OH due to the fact that the most positively charged area in MBE is adjacent to the H atom in the -OH group (Supplementary Fig. 19). The hydroxyl group dissociates. At the same time, the conjugated *π* orbitals of C=C bonds keep away from negatively charged Pd. These results are in good consistency with the IR spectra.

In addition, the interstitial atoms inhibit the diffusion of H to the subsurface and the formation of a highly active *β*-PdH_X_ phase, as verified by TPHD and DFT calculations. The electrochemical H adsorption experiment in the HClO_4_ solution also confirms this (Supplementary Fig. 20), from which the adsorption strength of H on Pd is qualitatively determined by the oxidation charge of the adsorbed hydrogen on the Pd surface. Pd/OMC shows obvious hydrogen adsorption-desorption peaks in the CV curve, indicating strong adsorption, while this peak disappears for Pd(B,C)/OMC. The absence of a *β*-PdH_X_ phase may be attributed to the inhibition of further hydrogenation of MBE over the B-modified Pd catalyst on which the desorption barrier of MBE is relatively low.

We report that the simultaneous increase in alkenol selectivity and alkynol conversion is achieved by manipulating interstitial atoms in Pd catalysts to gain *d* electron. However, the understanding of *p*-block dopants in Pd catalysts are still limited. Future works are expected to develop a feasible synthesis approach for metal nanocatalysts containing *p*-block elements with controllable compositions and ensemble structure by clarifying the atom diffusion, and experimentally identifying the compositions and atomic structure of active ensembles in nanocatalysts and their effects on the geometric and electronic structure of Pd.

In summary, ordered mesoporous carbon-supported interstitial Pd catalysts have been synthesized and provide ways to circumvent the linear scaling relationship in the semi-hydrogenation of MBY to MBE. The excellent activity (TOF value of 2.84 s^−1^), selectivity (95%), and stability of interstitial Pd(B,C)/OMC catalysts has been shown. By comparison, the pure Pd catalyst shows a high TOF value but a very low selectivity to MBE; and the Lindlar catalyst shows a high selectivity but the TOF value is pretty low (17 times lower than Pd(B,C)/OMC). The breaking of LSR is attributed to the rearrangement of MBE from an adsorption configuration using C=C bonds to one using the -OH terminations induced by the increase in the *d* charge of Pd. A linear relationship was established for the activation entropy between MBY and MBE according to the filling of the *d*-orbital of interstitial Pd catalysts. Our work not only provides a green and industrially available synthesis route for interstitial Pd nanocatalysts but also guides the rational design of industrial Pd catalysts with unique chemoselectivity in organic reactions.

## Methods

### Synthesis of Pd nanocatalysts with interstitial atoms encapsulated in ordered mesoporous carbonaceous carriers

Pd nanocatalysts with interstitial atoms encapsulated in ordered mesoporous carbonaceous carriers were synthesized using coordination-assisted and evaporation-induced self-assembly. The synthesis involves carbon precursors of low-molecular-weight phenolic resins (PF), a palladium source (PdCl_2_, >99 wt%, Shanghai Chemical Co.), a boron coordinating reagent (C_18_H_15_BO_3_, triphenyl borate, 99 wt%, Leyan.), a phosphorus coordinating reagent (C_18_H_15_O_3_P, triphenyl phosphite, 99 wt%, Macklin.), a carbon coordinating reagent (C_6_H_8_O_7_, citric acid, 99 wt%, Macklin.), a sulfur coordinating reagent (C_4_H_6_O_4_S, mercaptosuccinic acid, 98 wt%, Macklin.), a nitrogen coordinating reagent (C_4_H_5_N, Urea, 99 wt%, Adamas.), a structure-directing agent (poly(ethylene oxide)-b-poly(propylene oxide)-b-poly(ethylene oxide) triblock copolymer F127, EO_106_PO_70_EO_106_, MW = 12,600 g mol^−1^, Acros Chemical Inc.). In a typical synthesis, 1.0 g of F127 was first mixed in the presence of 20.0 g of ethanol for 10 min. Next, 1.1 mL of PdCl_2_ solution (palladium concentration in ethanol: 56.4 mmol L^−1^) was added to the first solution. At the same time, 5.0 g of low-molecular-weight phenolic resins (PF), and coordinating reagent (0.5 g triphenyl borate, 0.6 g triphenyl phosphite, 0.1 g citric acid, 1.0 g mercaptosuccinic acid, or 0.2 g urea.) were mixed together to obtain a clear solution. After 10 min, a solution containing 5.0 g of phenolic resins and a coordinating reagent was added dropwise to the first solution. The solution was stirred for 10 min at 40 °C, and then the mixture was transferred into evaporating dishes. The solvent evaporation was carried out in a hood at ambient temperature for 8 h, and the resins were polymerized in an oven at 100 °C for 24 h. The as-made films were scratched from the dishes and ground into fine powders. Calcination was carried out in a tubular furnace under nitrogen flow to obtain ordered mesoporous carbonaceous carriers supported Pd nanocatalysts with interstitial atoms including B, P, C, S, and N. The temperature program was from room temperature to 350 °C with a ramp of 1 °C min^−1^, and then maintenance for 5 h. The resulting catalysts were denoted as Pd (X,C)/OMC, X = B, P, C, S, N. Customized commercial Pd catalysts including Pd/C and Lindlar catalysts poisoned with lead were purchased from Aladdin.

### Characterization of the materials

The XRD measurements were taken using a Rigaku Dmax-3C diffractometer using Cu *K*α radiation (40 kV, 20 mA, *λ* = 0.15408 nm), The metallic Pd sizes were calculated according to the Scherrer formula, *D*_Pd_ = 0.89*λ*/*β*cosθ, on the basis of the (111) diffraction peak in the wide-angle XRD patterns. TEM images were recorded on a JEM 2100 microscope operating at 200 kV, The AC-STEM images were performed on a Hitachi HF5000 Environmental STEM operated at 200 kV equipped with a HAADF-STEM detector. The elemental analyses of the lines selected in the AC-HAADF-STEM images were performed using a Gatan 965 GIF Quantum instrument. The EELS were obtained in STEM mode with a focused electron beam in the sub-nanometer range. ^11^B solid-state NMR spectra was acquired at a static magnetic field strength of 14.1 T on a Bruker Advance III spectrometer, operating at a ^11^B Larmor frequency of 192.50 MHz with a commercial triple-resonance 1.9 mm MAS probe. Spectra were referenced to NaBH_4_ at −42.06 ppm. The TPHD experiments were conducted with a Micromeritics Auto Chem II 2920 instrument. 100 mg of catalyst was first reduced in a quartz reactor under a 10 vol.% H_2_/Ar with a flow rate of 30 cm^3^ min^−1^ at a temperature of 100 °C for 2 h. Then the reactor was cooled to 30 °C under the same H_2_/Ar flow to avoid thermal decomposition of the Pd-H phase, and it was further flushed with Ar flow (30 cm^3^ min^−1^) for 30 min to remove weakly adsorbed hydrogen. After that, it was heated to 150 °C at a temperature ramp of 5 °C min^−1^ under 10 vol.% H_2_/Ar with the same flow rate of 30 cm^3^ min^−1^. The spilled hydrogen in this process is monitored by a thermal conductivity detector (TCD). CO-DRIFTS was measured on a Thermo Scientific Nicolet iS50 spectrometer equipped with an in situ cell (PIKE DiffusIR, KBr windows) and a liquid-N_2_-cooled mercury cadmium telluride (MCT) detector with CO as a probe molecule. the catalysts were firstly dried in a vacuum at 100 °C for 1 h followed by a reduction in 10 vol.% H_2_/Ar (30 cm^3^ min^−1^) for 60 min at the same temperature. After the sample was allowed to cool down to room temperature in argon (30 cm^3^ min^−1^), background spectra were acquired and the material was subsequently exposed to diluted CO (5 vol.% CO/Ar, 30 cm^3^ min^−1^) for 15 min. Spectra were acquired in the range of 4000–650 cm^−1^ with a resolution of 4 cm^−1^ after flushing with Ar (20 cm^3^ min^−1^) until no signal for gas-phase CO was detected. For in situ FT-IR adsorbing MBE, the catalysts were firstly dried in a vacuum and reduced to 10 vol.% H_2_/Ar (30 cm^3^ min^−1^) for 1 h at 100 °C. After an initial background scan, the liquid adsorbate (removed water by MgSO_4_) was introduced by bubbling with an Ar flow (50 cm^3^ min^−1^) to the in situ cell. After saturation exposure, the Ar flow was also used to fully purge the gas-phase adsorbate. During the experiments, the spectra were also collected in the range 4000–650 cm^−1^ with a resolution of 4 cm^−1^_._ The XPS information was performed on a Perkin-Elmer PHI 5000 CESCA instrument with a base pressure of 10^−9^ Torr. The samples were evacuated in a loading locked chamber and then transferred to the analysis chamber (10^−9^ mbar). The measured XPS data was calibrated with a C 1 *s* binding energy of 284.6 eV. The XANES measurement for the Pd *L*_3_-edge (3173 eV) were performed in fluorescence mode on beamline 9-BM-B with electron energy of 7 GeV and an average current of 100 mA which is located in the Advanced Photon Source at Argonne National Laboratory. The radiation was monochromatized by a Si(111) double-crystal monochromator. The data processing were performed using the Athena program.

### Computational methods

The Vienna Ab-initio Simulation Package (VASP)^55,56^ was performed to evaluate the adsorption and hydrogenation mechanism. The Pd(111) surface was modeled using a supercell of (5 × 5) with five atomic layers. The bottom three layers were fixed at the bulk position during the geometry optimization. A vacuum layer of 20 Å was used to avoid interaction between neighboring slabs. The generalized gradient approximation (GGA) with the Perdew–Burke–Ernzerhof (PBE) functional was used to describe the electronic exchange and correlation effects^57^. A plane wave basis sets with a cutoff energy of 500 eV was used to expand the solution of the Kohn-Sham equations. The Brillouin zone was sampled by a 4 × 4 × 1 Monkhorst-Pack *k*-point grid^58^. The geometry optimization converged to a force on each atom less than 0.03 eV Å^−1^ and the total energy smaller than 10^−6^ eV. The surface and subsurface *p*-block atoms modified surfaces were modeled by optimizing the position of the non-metal atoms on the surface and subsurface, respectively. In our systems, a face center cubic Pd(111) surface was chosen as the model base on our XRD analysis. The surface non-metal atoms preferred to locate at the trigonal hollow *hcp* site of Pd(111), while the subsurface non-metal atoms preferred to locate at the octahedral subsurface sites of the Pd lattice. These results were in good agreement with the previous studies^41,42,59^. The surface sites referred to the *hcp* sites of Pd(111), while the subsurface sites referred to the octahedron sites of Pd(111). The adsorption energies of non-metal atoms on the Pd(111) surface were calculated as follows:

1 $${E}_{{{{{{\rm{ads}}}}}}}={E}_{{{{{{\rm{Pd}}}}}}/{{{{{\rm{adsorbate}}}}}}}-{E}_{{{{{{\rm{Pd}}}}}}}-{E}_{{{{{{\rm{adsorbate}}}}}}}$$

where *E*_Pd/adsorbate_ is the total energy of the relaxed adsorbate-surface system, while *E*_Pd_ and *E*_adsorbate_ are the total energy of the relaxed bare surface and gas-phase adsorbate, respectively^60^. Hence, the adsorption energy is defined as negative if the total energy decreases when the adsorbate was brought from infinity and placed onto the surface. The transition states (TSs) were searched using a method called a climbing image nudged elastic band (CI-NEB) optimization scheme^61^. The transition states were confirmed by two rules: (1) all forces on atoms have been optimized to be less than 0.03 eV Å^−1^; (2) the total energy is a maximum along the reaction coordinate but a minimum with respect to all other degrees of freedom. Vibrational frequency analyses were performed to confirm the integrity of the initial states, transition states, and final states.

The Gaussian 09 software^62^ was used to obtain the theoretical IR spectra. The geometry optimization and frequency/intensity calculations of MBE on the surface of the Pd_7_ plane were performed by applying the Becke exchange functional and the Lee-Yang-Parr correlation functional (BLYP). The basis set used for C, N, H atoms is the 6–311 + G(*d,p*), and the pseudopotential basis sets Lanl2DZ was used for the Pd atoms. The tight convergence criterion of Gaussian 09 was used in structure optimization, and the ultrafine integration grid was used in the numerical integration of the structure optimization and vibrational frequencies calculation. The geometries of MBE-Pd_7_ were fully optimized without imaginary frequencies. The calculations of relative FT-IR spectra with a resolution of 8 cm^−1^ were carried out at the same level of theory using the same basis set. The scaling factor for the harmonic vibration frequencies of BLYP/6–311 + G(*d,p*) is 1.0001^63^. Vibrational mode assignments, molecular orbital, and electrostatic potential were made by the Gaussview program 5.0.8 version^64^.

### Catalytic performance tests

Hydrogenation reactions of MBY or MBE were performed in a 10 mL round-bottom flask filled with a balloon containing 100% H_2_. In a typical reaction, 125 μL of MBY or 131 μL of MBE and 5 mL of ethanol, 8 mg of powdered Pd(B,C)/OMC catalyst were placed in the round-bottom flask. Then the air in the round-bottom flask was replaced by 100% H_2_ three times and the flask was then sealed by an atmospheric balloon full of 100% H_2_, the stirring speed was fixed at 800 rpm and the temperature was kept at 25 °C. After each reaction, the catalyst was separated by filtration and rinsed with 5 mL of ethanol. The filtrate was identified and analyzed to determine the conversion and yield by gas chromatography spectrometry (GC) (Agilent 7890B) using an HP-INNOWax capillary column. The MBY or MBE hydrogenation was repeated at least three times with ±5% experimental errors (standard deviation). The carbon balance for all tests was above 97%. The reaction with deuterium gas was the same as above with the hydrogen replaced by D_2_. The catalytic results are shown in terms of the conversion of MBY, selectivity of MBE, the initial reaction rate (molecules of reactants converted per mole of Pd per second), and the turn-over frequency (TOF, molecules of reactants converted per surface atom of Pd per second). The TOF value was calculated at a conversion below 20%. The exposed surface area of the dispersed Pd nanoparticles was estimated by pulsing CO chemisorption.

The TOF, *E*_a_, and Δ*S*^0*^ for the hydrogenation reactions of MBY and MBE overall studied Pd catalysts were obtained and are described in the Supplementary Information.

### Mass transfer limitation tests

The external mass transfer limitations were studied by ranging stirring speeds from 700 to 1000 rpm. The internal diffusion effect on the reaction rate was studied by controlling the surface concentrations of palladium but with similar particle size, known as the Madon–Boudart (MB) test.

### Hot filtration tests

During the reaction, the hot filtration test during the reaction was performed after achieving 50–60% MBY conversion and then removing the solid catalyst under hot conditions. Then further stirred for 0.5–1 h. The liquid phase was analyzed by GC to determine the change of MBY conversion.

### Recycling tests

For the recycling study, the MBY was hydrogenated under the same conditions as previously described. After each reaction, the catalyst was recovered by washing thoroughly with large amounts of ethanol and water. The recovered catalyst was further dried at 60 °C under vacuum overnight, weighed, and reused. To ensure that the same catalyst amount was used in each cycle, several parallel reactions were also carried out at the same time, except using the recovered catalyst. The reused catalyst was designated as Pd(B,C)/OMC-Ry_,_ wherein y was catalytic run. For example, Pd(B,C)/OMC-R8 was the Pd(B,C)/OMC catalyst after eight catalytic runs. The solutions after each run were collected and combined to determine the metal leaching.

### Trapping tests

Thiol group modified mesoporous silica (SH-SBA-15) was used to trap the soluble palladium species that had leached into the solution. In a standard experiment, 12 mg of SH-SBA-15 was placed in a reaction flask prior to the addition of the reaction solution to give a molar ratio of SH:Pd ≈30:1.

## Supplementary information

Supplementary Information

## Data Availability

The data that support the findings of this study are available from the corresponding author upon reasonable request.
