# Supplementary material for: Breaking scaling relationships in alkynol semi-hydrogenation by manipulating interstitial atoms in Pd with d-electron gain
Source: Nat Commun. 2022 May 18;13:2754. doi: 10.1038/s41467-022-30540-z (PMC9117217; doi:10.1038/s41467-022-30540-z)
Supplement: Supplementary file 1 — Supplementary Information [file 41467_2022_30540_MOESM1_ESM.pdf]

Supplementary Information

Breaking scaling relationships in alkynol semi-hydrogenation by manipulating interstitial atoms in Pd with *d*-electron gain

Yang Yang[1#], Xiaojuan Zhu[1#], Lili Wang[1#], Junyu Lang[2], Guohua Yao[1], Tian Qin[3], Zhouhong Ren[3], Liwei Chen[3,4], Xi Liu[3*], Wei Li[5*], Ying Wan[1*]

[1]The Education Ministry Key Laboratory of Resource Chemistry, Joint International Research Laboratory of Resource Chemistry of Ministry of Education, Shanghai Key Laboratory of Rare Earth Functional Materials, and Shanghai Frontiers Science Center of Biomimetic Catalysis, Shanghai Normal University, Shanghai 200234, China.

[2]School of Physical Science and Technology, Shanghai Tech University, Shanghai 201210, China.

[3]School of Chemistry and Chemical Engineering, In-situ Center for Physical Sciences, Shanghai Jiao Tong University, Shanghai 200240, China.

[4]School of Chemistry and Chemical Engineering, Frontiers Science Center for Transformative Molecules, Shanghai Jiao Tong University, Shanghai 200240, China.

[5]Department of Chemistry, Laboratory of Advanced Materials, Shanghai Key Laboratory of Molecular Catalysis and Innovative Materials, iChEM and State Key Laboratory of Molecular Engineering of Polymers, Fudan University, Shanghai 200433, China.

[#]These authors were equal major contributors: Yang Yang, Xiaojuan Zhu and Lili Wang.

[*]Correspondence and requests for materials should be addressed to X.L. (email: liuxi@sjtu.edu.cn); W.L. (email: weilichem@fudan.edu.cn); Y.W. (email: ywan@shnu.edu.cn)

Contents

Supplementary Figures 3

Supplementary Tables 23

Supplementary Methods 26

Supplementary References 30

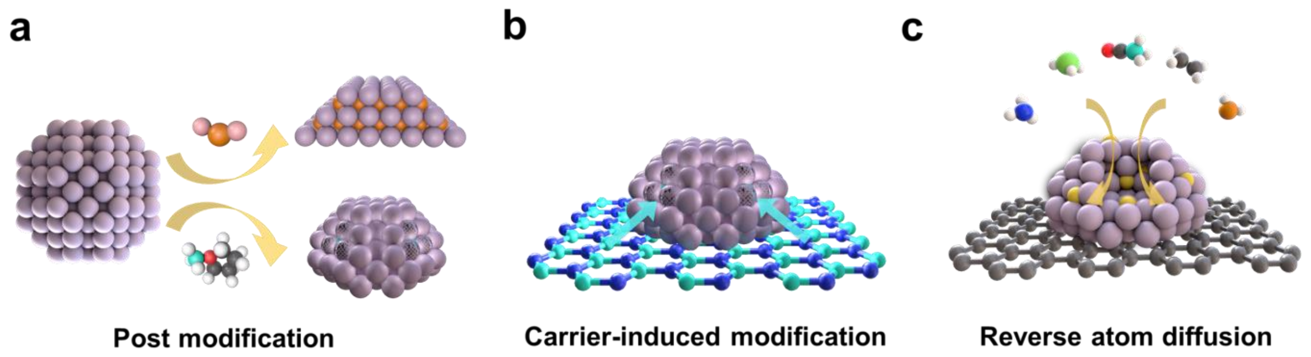

Supplementary Fig. 1. Schematic of Pd modified with *p*-block elements. (a) The post modification using Na_2S or borane tetrahydrofuran (BH_3.THF) in liquid phase. **(b)** The B atoms from defects in porous boron nitride (PBN) to the Pd subsurface. **(c)** The reverse atom diffusion by atom deposition and diffusion from released gas molecules (BH_3CO, PH_3, C_2H_2, H_2S, and NH_3) into the Pd lattice. Pd purple, Na pink, H white, B light cyan, C dark gray, O red, S orange, P green.

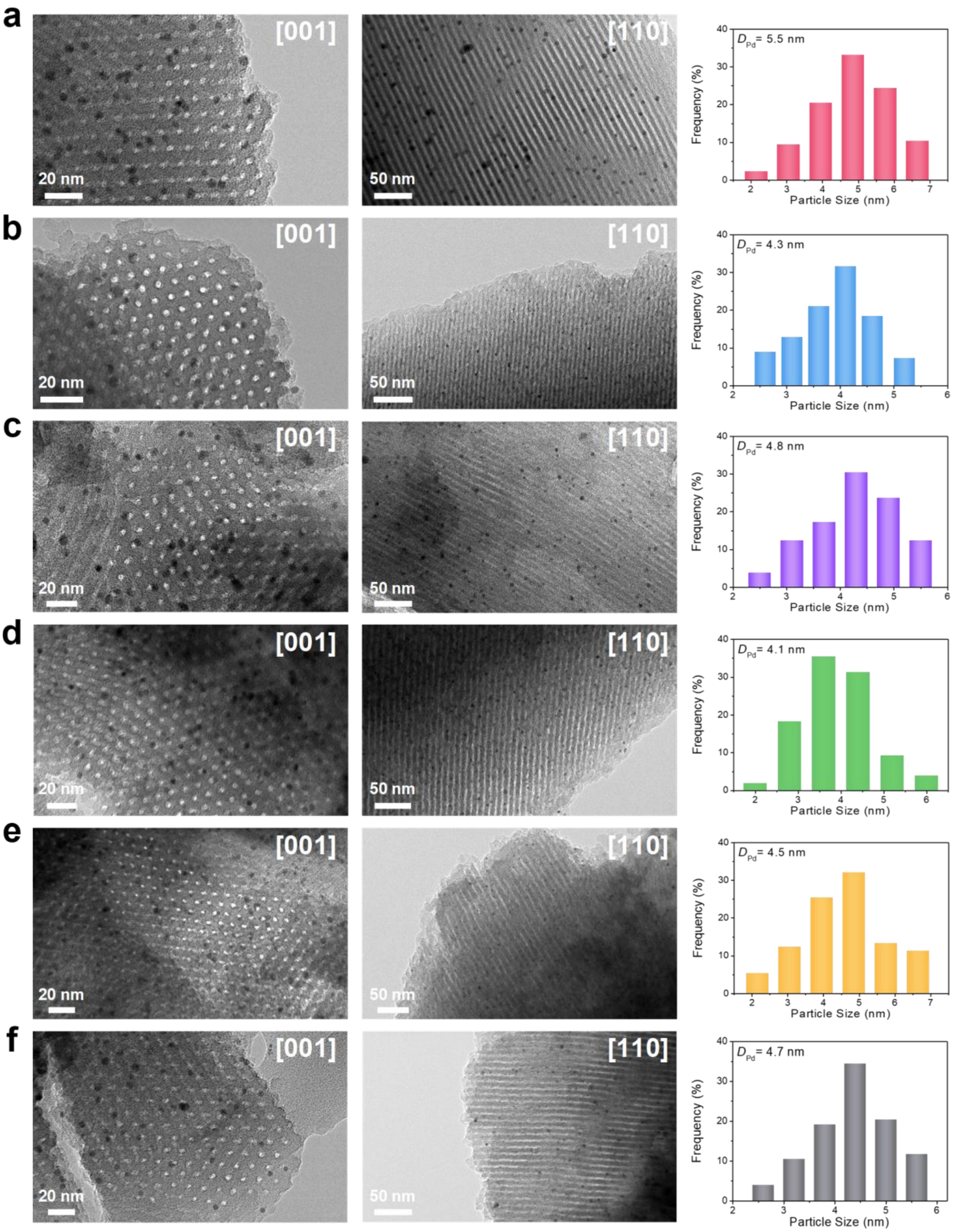

Supplementary Fig. 2. Transmission electron microscope (TEM) images. TEM viewed along the [001] and [110] directions for Pd interstitial nanocatalysts and reference catalyst: **(a)** Pd(B,C)/OMC, **(b)** Pd(P,C)/OMC, **(c)** Pd(C)/OMC, **(d)** Pd(S,C)/OMC, **(e)** Pd(N,C)/OMC and **(f)** Pd/OMC. The particle size distribution obtained by measuring at least 200 nanoparticles.

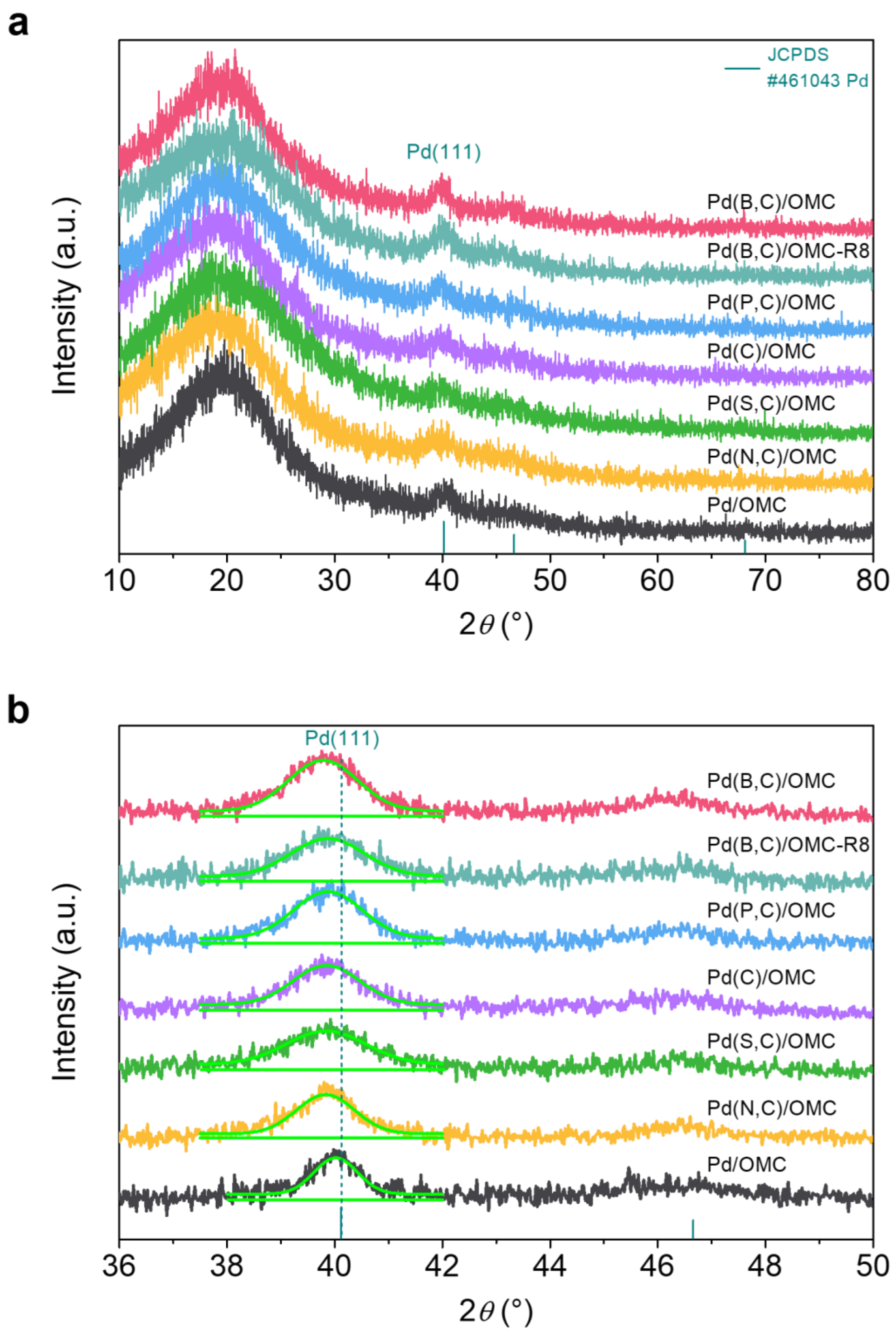

Supplementary Fig. 3. Wide-angle X-ray diffraction (XRD) patterns. (a) A scan of 5° min^{-1} collected from 10 to 80° and **(b)** a fine scan of 0.4° min^{-1} containing higher statistics collected near the diffraction peak at ~40° for Pd interstitial nanocatalysts with different interstitial atoms and the Pd/OMC reference catalyst without interstitial atoms. Pd(B,C)/OMC-R8 is the Pd(B,C)/OMC catalyst after eight catalytic runs.

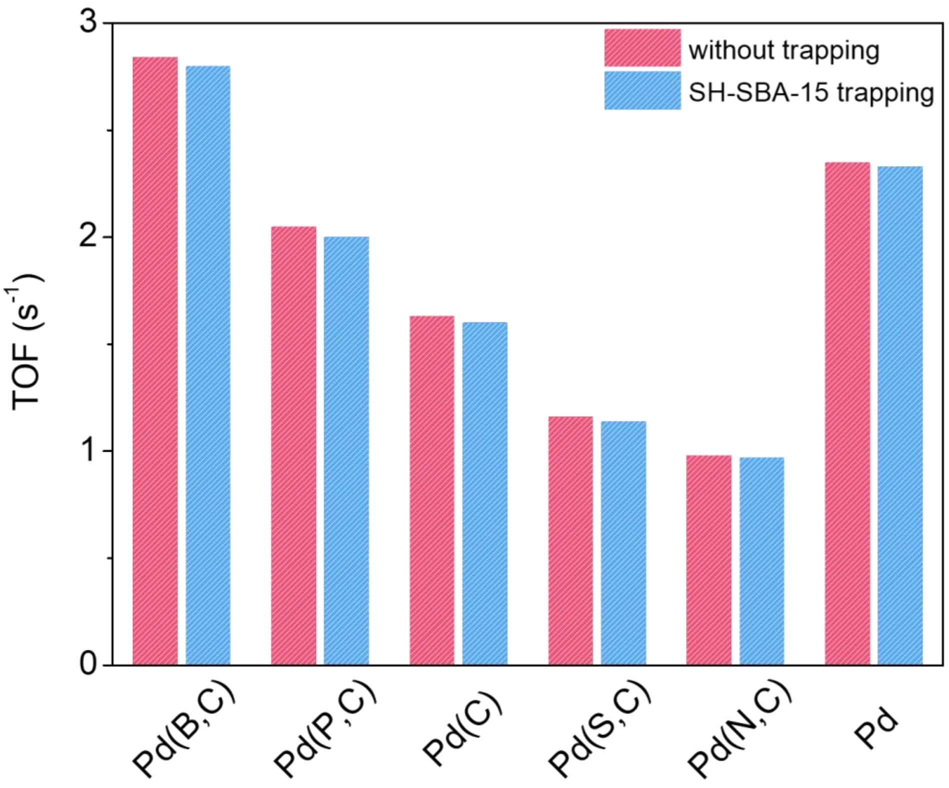

Supplementary Fig. 4. Trapping tests. Comparison of the turn-over frequency (TOF) value of the hydrogenated 2-methyl-3-butyn-2-ol (MBY) between the reaction and reaction in the presence of a solid SH-SBA-15 trapping agent over various Pd catalysts (S:Pd ≈ 30 in molar ratio) at the conversion of approximately 20%. The reaction conditions were: 0.045 mol% Pd; 1.25 mmol of substrate; 5 mL of ethanol; 25 °C; 800 rpm; and in the presence of hydrogen by an H_2 balloon under atmospheric pressure.

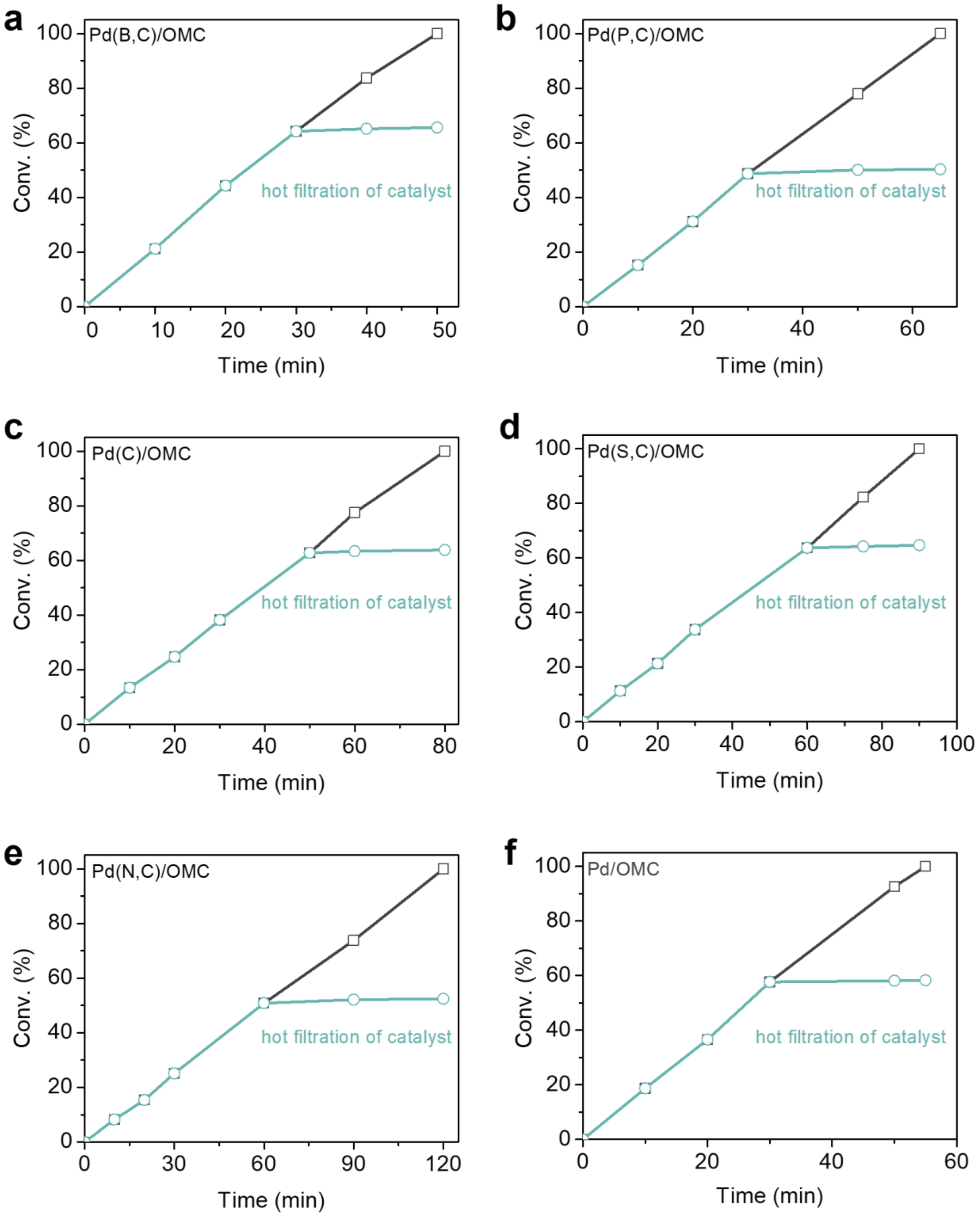

Supplementary Fig. 5. Hot filtration experiments. All studied Pd interstitial nanocatalysts **(a-e)** and the reference Pd/OMC catalyst **(f)** were removed at the conversion of approximately 50-60%, under reaction temperature 25 °C (light cyan line). For comparison, the conversion plot for the MBY over Pd nanocatalysts along with the reaction time is also provided (black line). The reaction conditions were: 0.045 mol% Pd; 1.25 mmol of substrate; 5 mL of ethanol; 25 °C; 800 rpm; and in the presence of hydrogen by an H_2 balloon under atmospheric pressure.

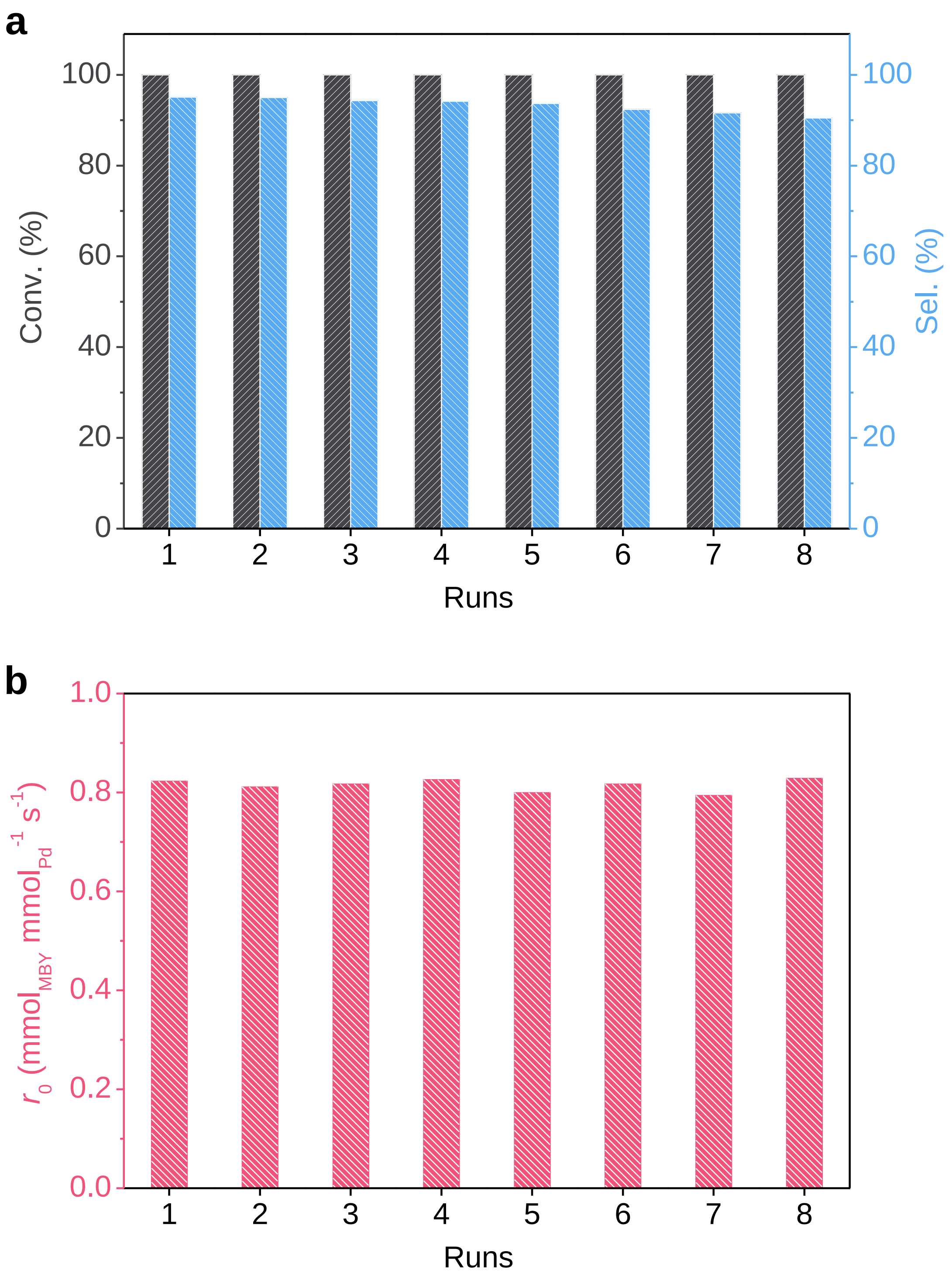

Supplementary Fig. 6. Reusability. The comparison of the **(a)** overall conversion and selectivity, and **(b)** the initial reaction rate (r_0) for the Pd(B,C)/OMC catalyst in the successive cycles for the semi-hydrogenation of MBY. The reaction conditions were: 0.045 mol% Pd; 1.25 mmol of substrate; 5 mL of ethanol; 25 °C; 800 rpm; and in the presence of hydrogen by an H_2 balloon under atmospheric pressure.

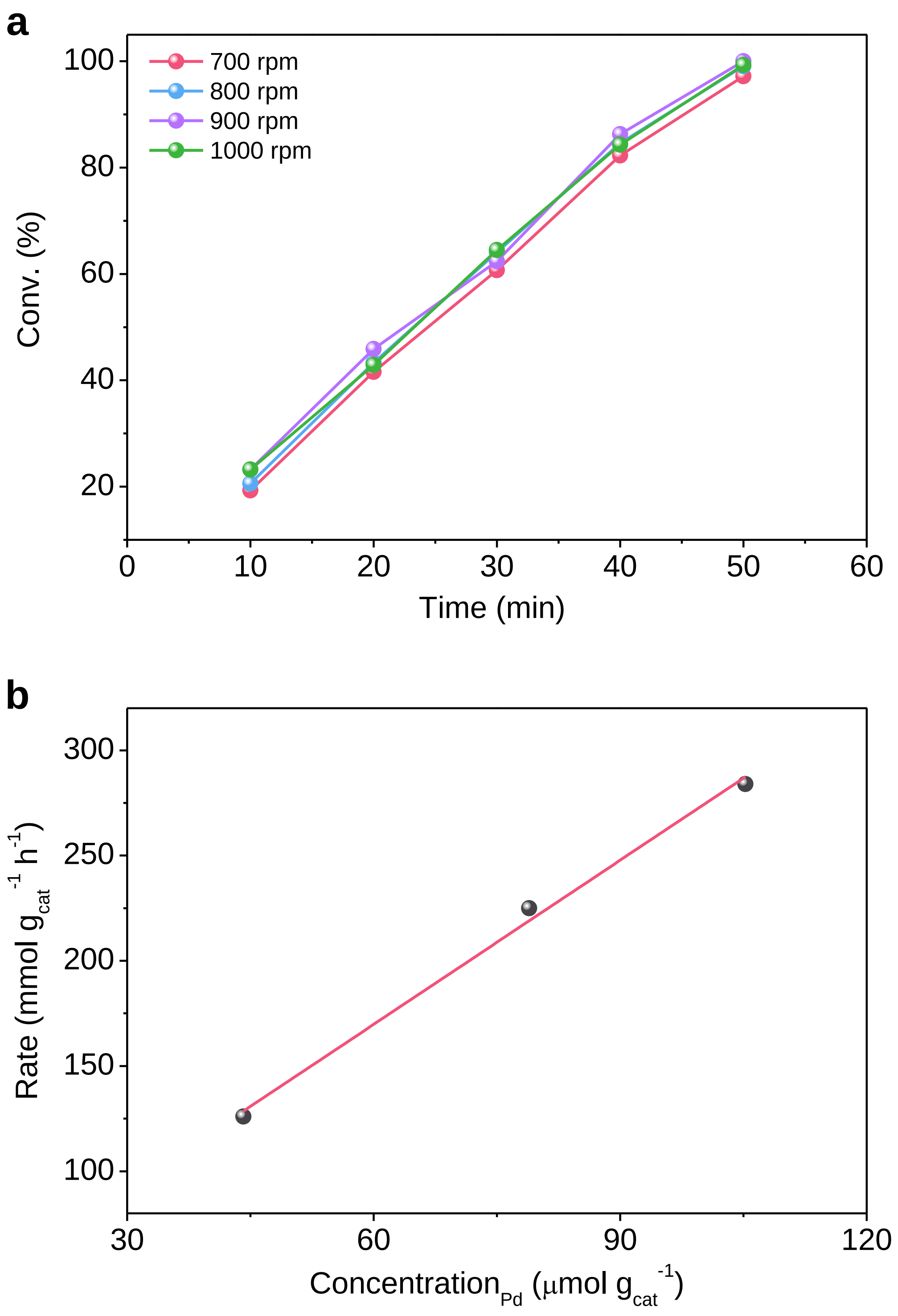

Supplementary Fig. 7. Mass transfer limitation tests. (a) Effect of the stirring rate on the conversion of semi-hydrogenation of MBY using the Pd(B,C)/OMC catalyst. The reaction conditions were: 0.045 mol% Pd; 1.25 mmol of substrate; 5 mL of ethanol; 25 °C; 700-1000 rpm; and in the presence of hydrogen by an H_2 balloon under atmospheric pressure. **(b)** The Madon-Boudart (MB) test. Effect of the Pd loading on the reaction rate of semi-hydrogenation of MBY using the Pd(B,C)/OMC catalyst. The reaction conditions were: 8 mg of catalyst; 1.25 mmol of substrate; 5 mL of ethanol; 25 °C; 800 rpm; and in the presence of hydrogen by an H_2 balloon under atmospheric pressure.

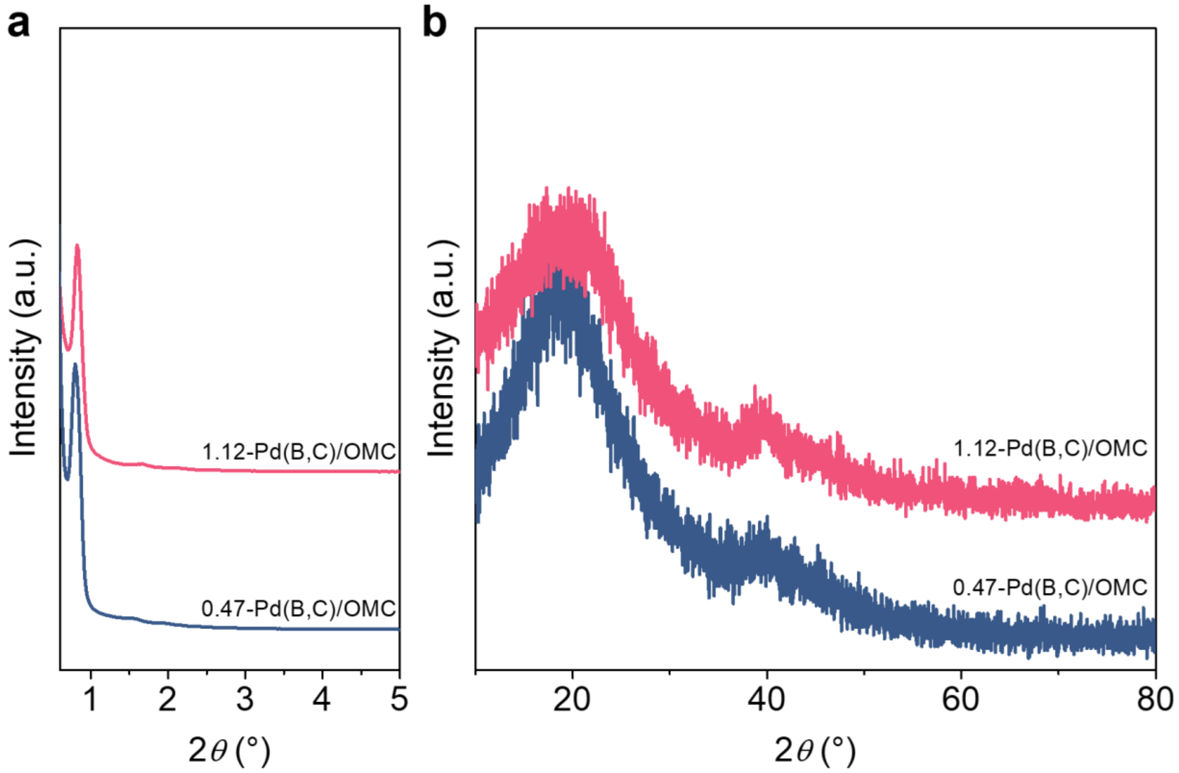

Supplementary Fig. 8. XRD analysis. (a) Small-angle XRD patterns and **(b)** wide-angle XRD patterns of Pd(B,C)/OMC catalysts with metal contents of 0.47 wt% and 1.12 wt%.

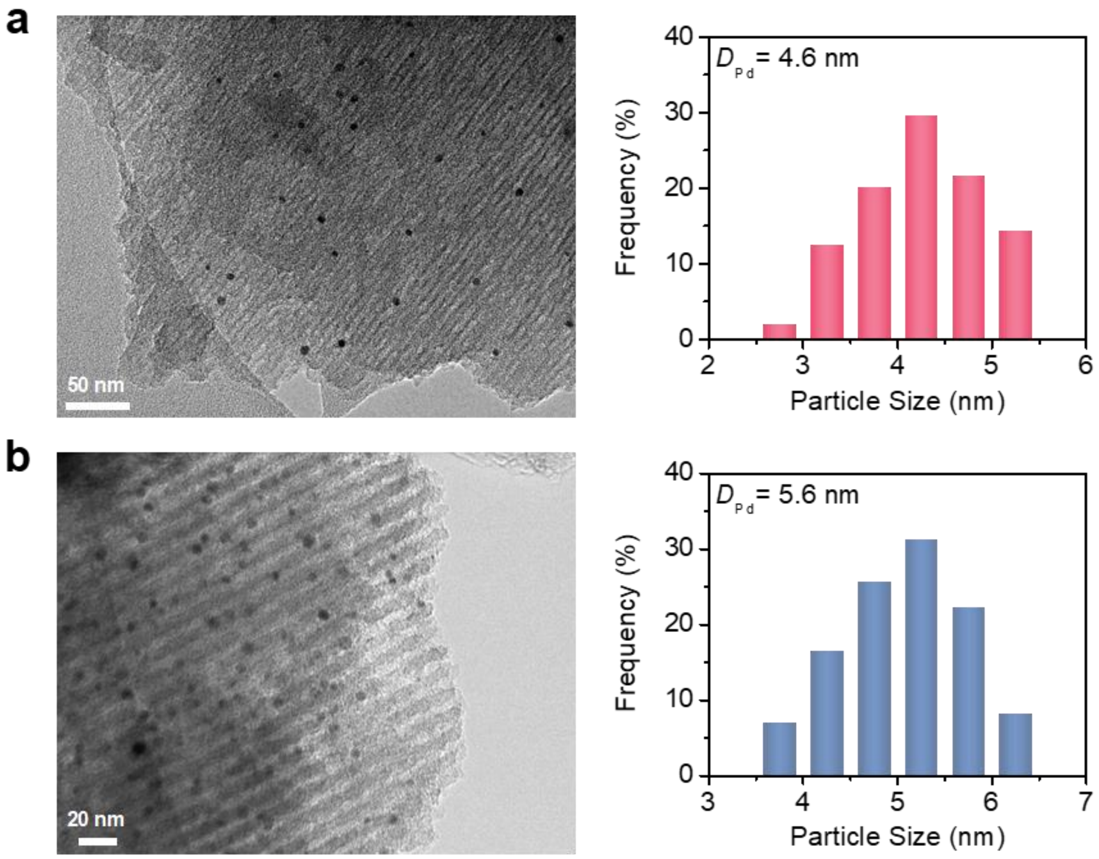

Supplementary Fig. 9. Particle size analysis. TEM images viewed along the [110] direction and the corresponding Pd particle size distribution of Pd(B,C)/OMC catalysts with total metal contents of **(a)** 0.47 wt% and **(b)** 1.12 wt%.

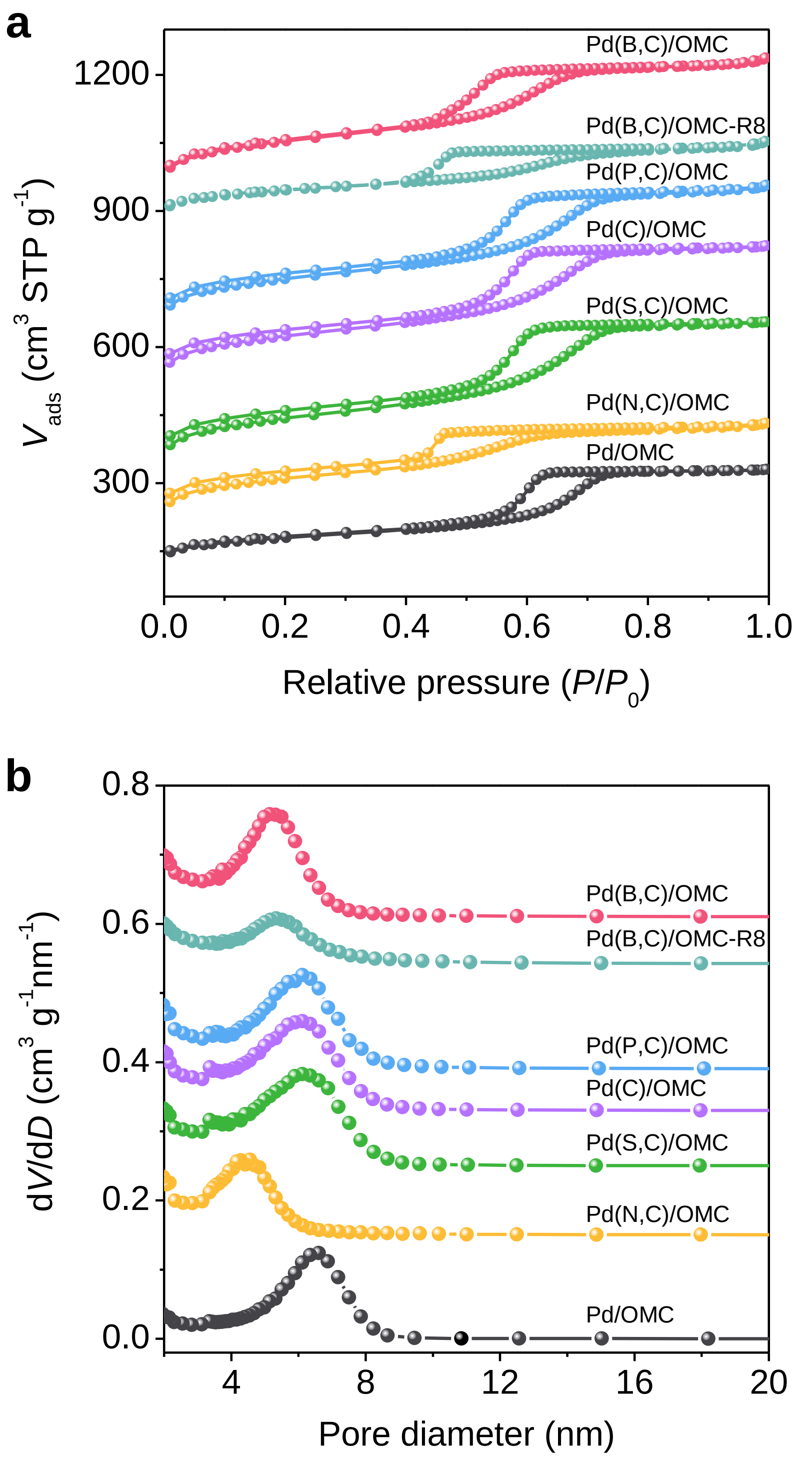

Supplementary Fig. 10. Pore structure. (a) N_2 sorption isotherms and **(b)** pore size distribution curves for Pd interstitial nanocatalysts and the Pd/OMC reference catalyst. Pd(B,C)/OMC-R8 is the Pd(B,C)/OMC catalyst after eight catalytic runs. The sorption isotherms are vertically offset by 200, 300, 500, 600, 800 and 850 cm^3 g^{-1} from Pd(N,C)/OMC to Pd(B,C)/OMC. The pore size distribution curves are vertically offset by 0.15, 0.25, 0.35, 0.40, 0.55 and 0.60 cm^3 g^{-1} nm^{-1} from Pd(N,C)/OMC to Pd(B,C)/OMC. Type-IV N_2 sorption isotherms were detected for all the catalysts studied, typical of the open mesoporous solids with uniform pore sizes. The pore sizes, Brunauer-Emmet-Teller (BET) surface areas, and pore volumes are approximately 6 nm, 500 m^2 g^{-1} and 0.5 cm^3 g^{-1}, respectively.

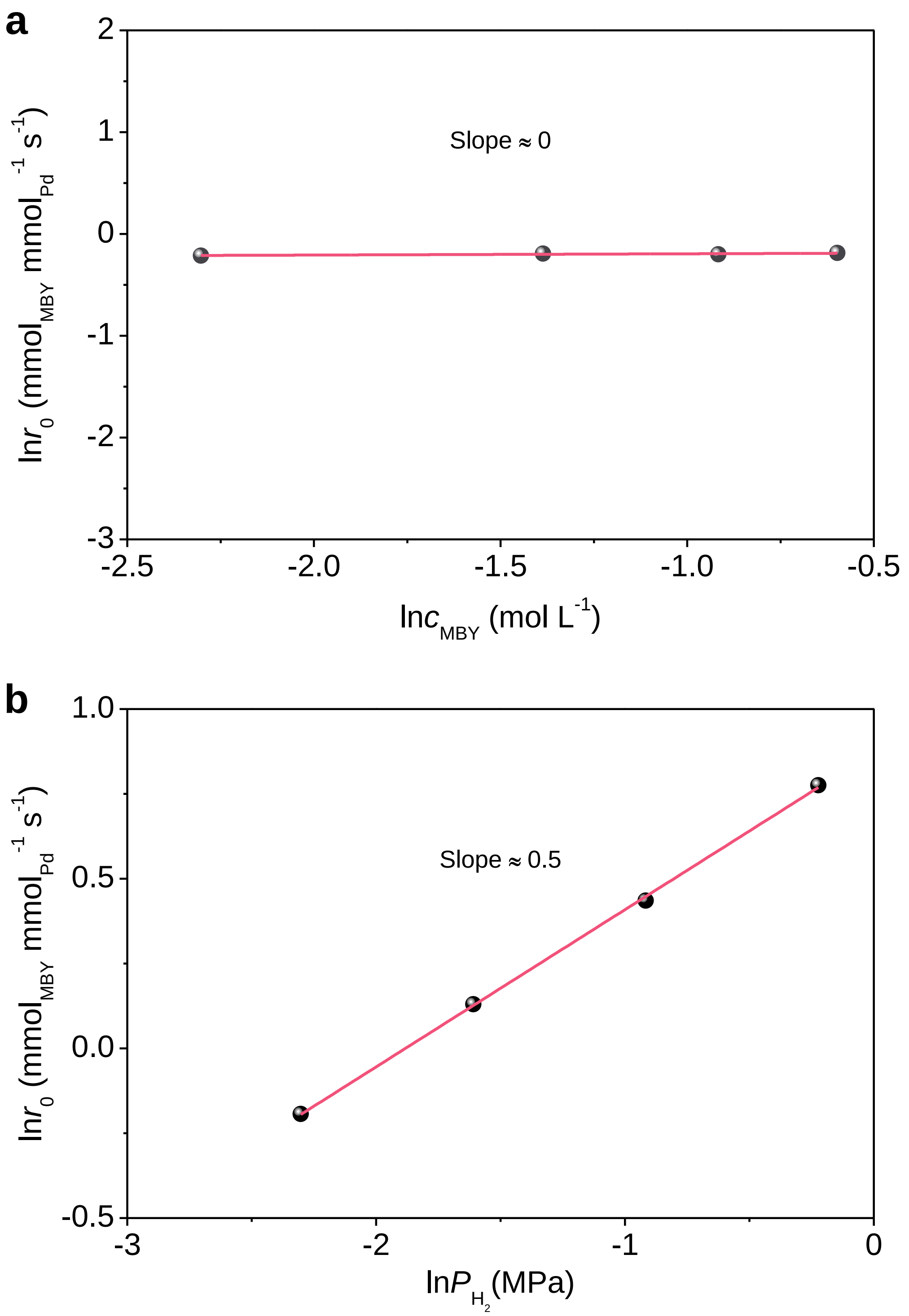

Supplementary Fig. 11. Reaction order. (a) Effect of MBY concentration on the initial reaction rate (r_0) of semi-hydrogenation over the Pd(B,C)/OMC catalyst. The reaction conditions were: 8 mg Pd(B,C)/OMC; 5 mL of ethanol; 25 °C; 800 rpm; and in the presence of hydrogen by an H_2 balloon under atmospheric pressure. **(b)** Effect of H_2 pressure on the initial reaction rate (r_0) of semi-hydrogenation over the Pd(B,C)/OMC catalyst. The reaction conditions were: 8 mg Pd(B,C)/OMC; 1.25 mmol of substrate; 5 mL of ethanol; 25 °C; 800 rpm.

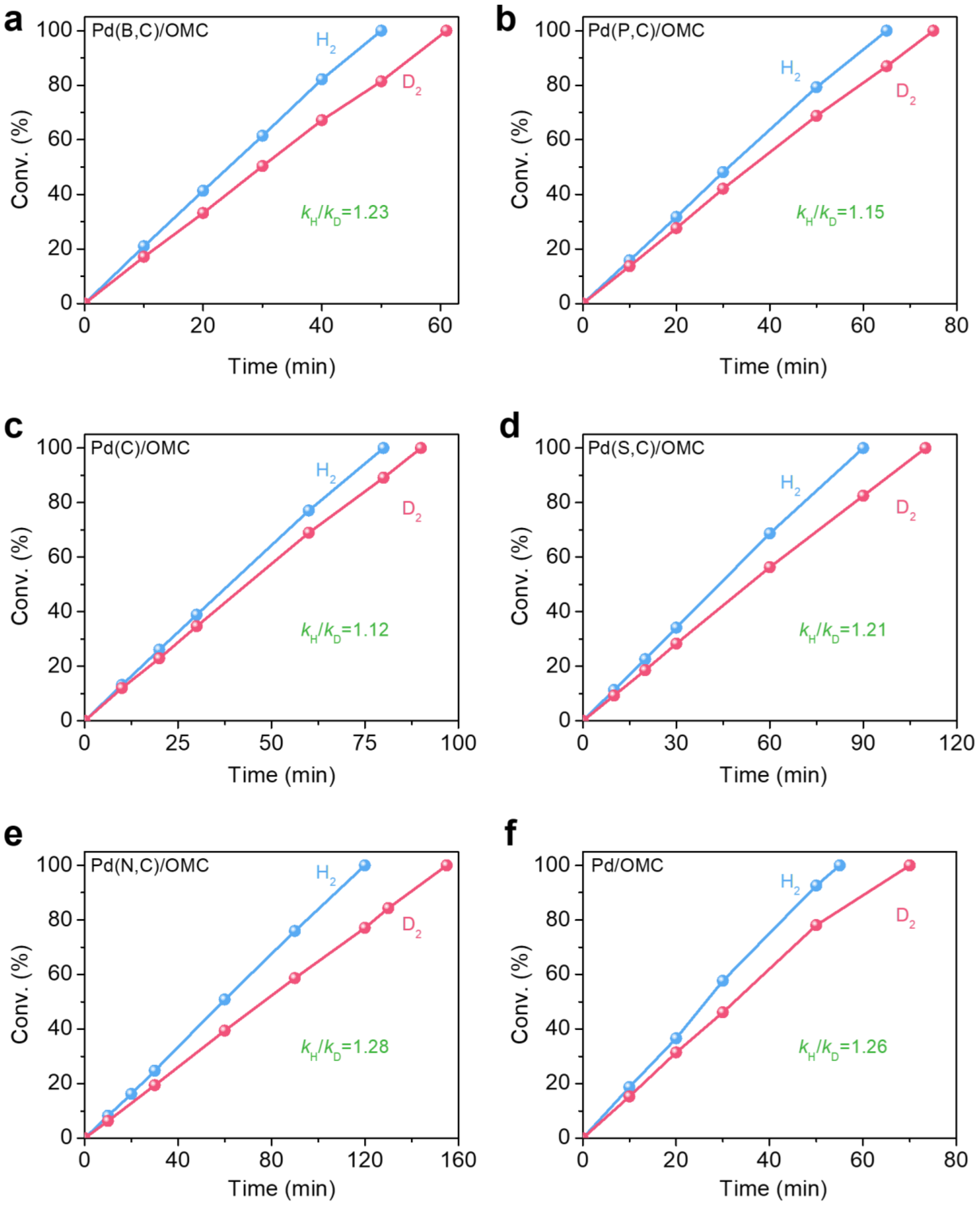

Supplementary Fig. 12. Kinetic isotope effect (KIE) experiments. KIE observed for all studied Pd interstitial nanocatalysts **(a-e)** and the reference Pd/OMC catalyst **(f)** in semi-hydrogenation of MBY. The reaction conditions were: 0.045 mol% Pd; 1.25 mmol of substrate; 5 mL of ethanol; 25 °C; 800 rpm; and in the presence of hydrogen/deuterium by an H_2/D_2 balloon under atmospheric pressure.

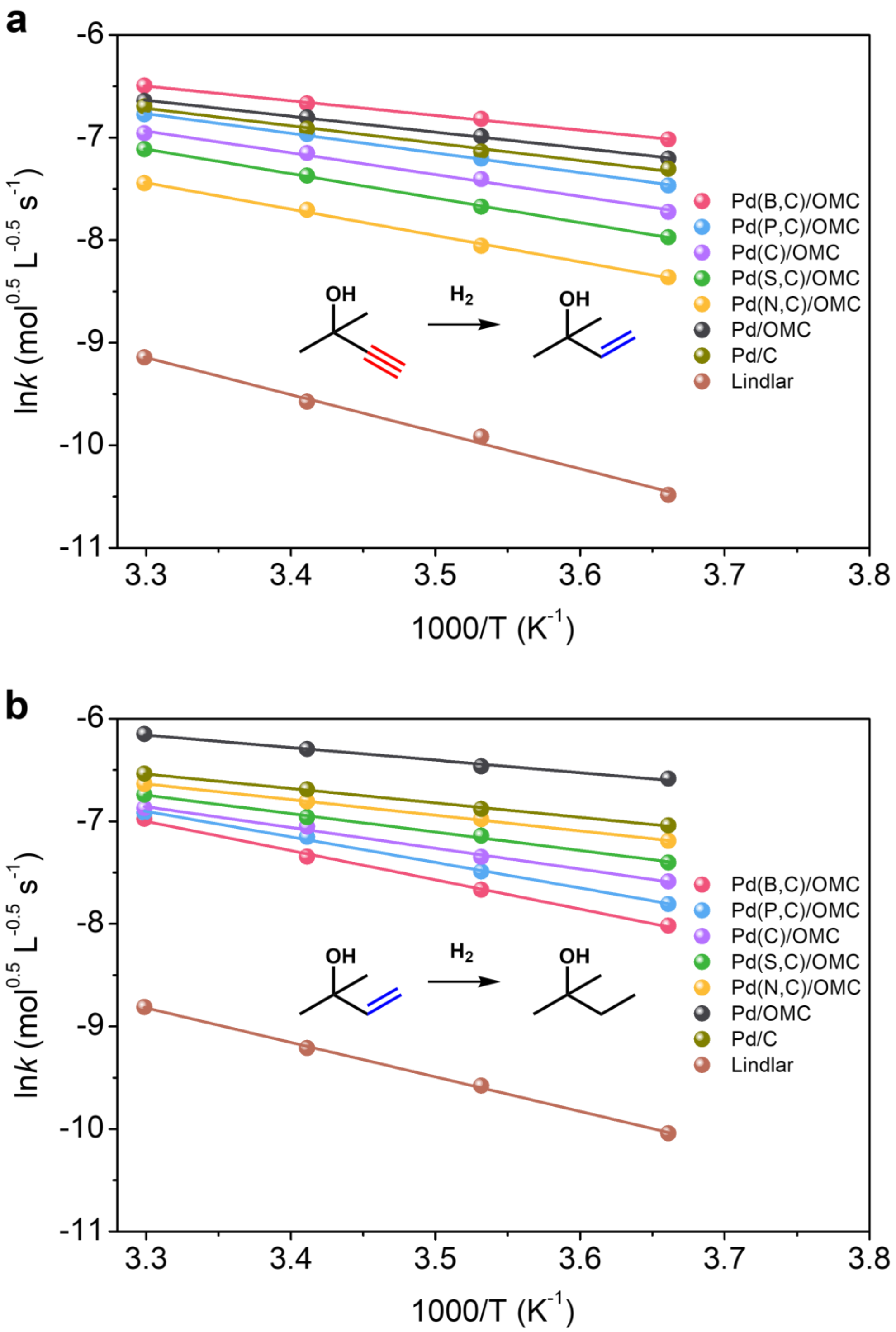

Supplementary Fig. 13. Arrhenius plots. (a) MBY hydrogenation and **(b)** 2-methyl-3-buten-2-ol (MBE) hydrogenation over Pd interstitial nanocatalysts. For comparison, the reference Pd/OMC, commercial Pd/C and Lindlar catalysts are also provided.

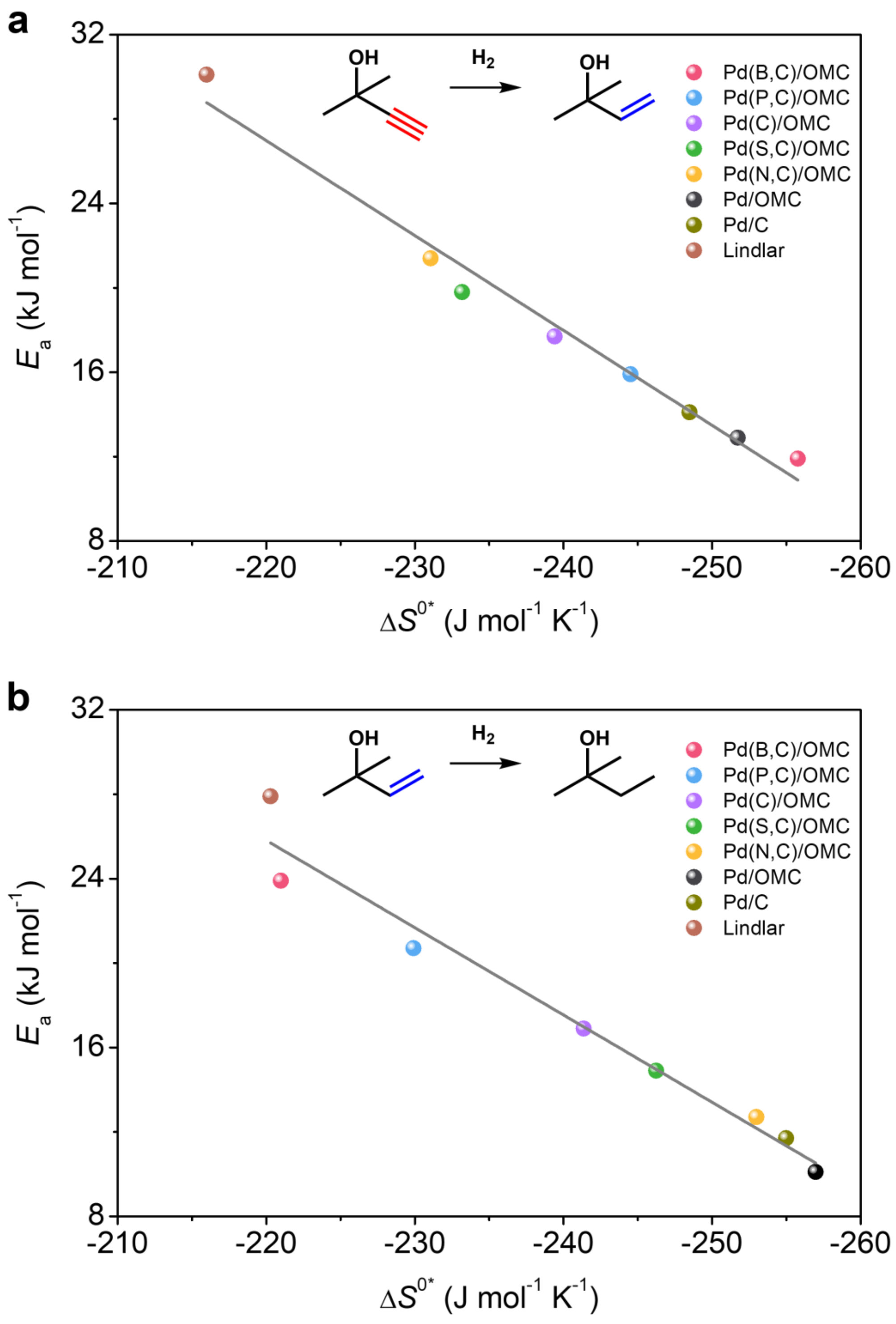

Supplementary Fig. 14. Plot of activation energy (E_a) against activation entropy (ΔS^{0*}). (a) MBY hydrogenation and **(b)** MBE hydrogenation over Pd interstitial nanocatalysts. For comparison, the reference Pd/OMC, commercial Pd/C and Lindlar catalysts are also provided.

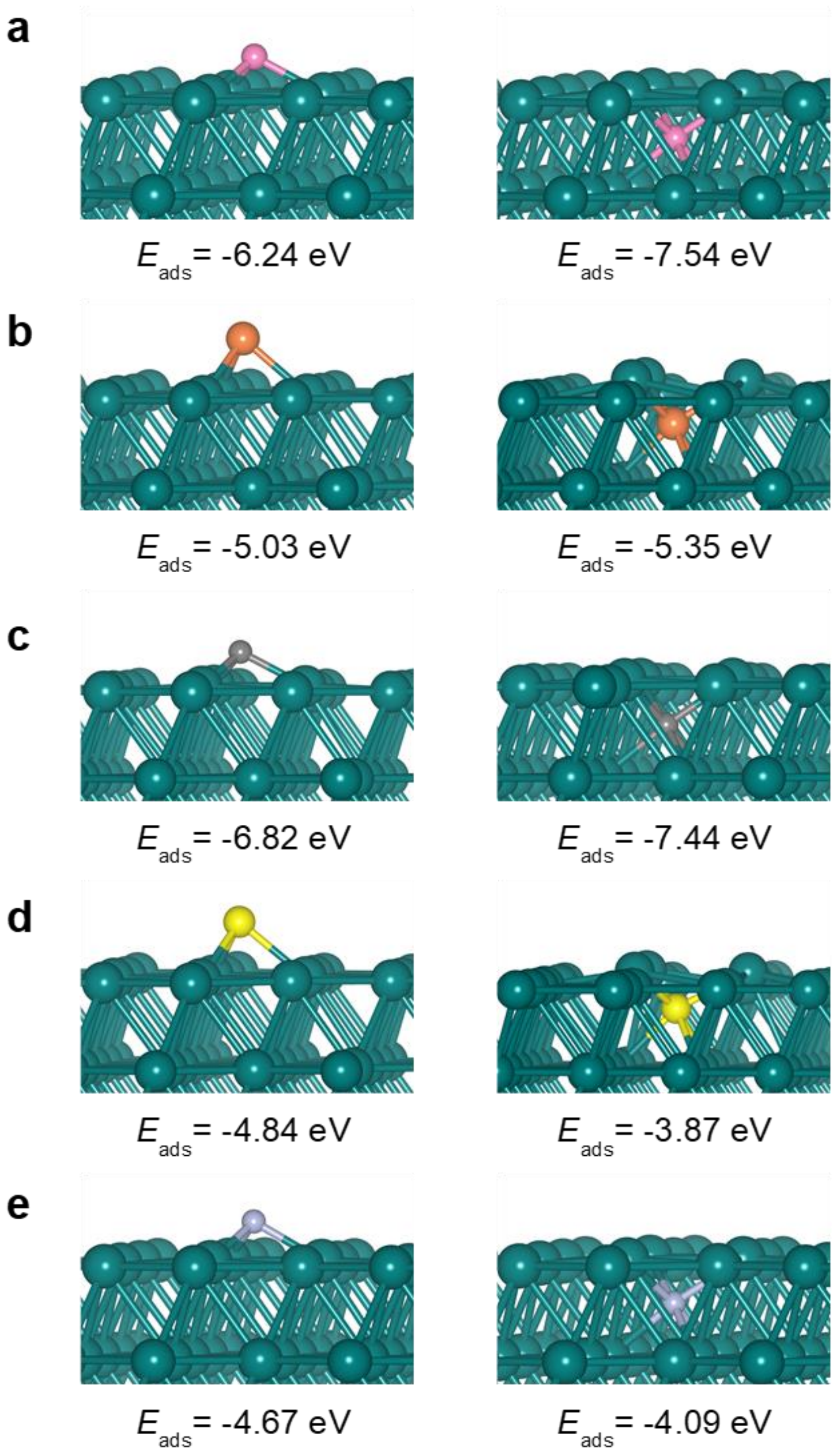

Supplementary Fig. 15. DFT calculation model. Optimized atomic structures of an adsorbed atom at the surface (*hcp* sites, up row) and subsurface (octahedral sites, below row) of Pd(111): (**a**) boron, **(b)** phosphorus, **(c)** carbon, **(d)** sulfur and **(e)** nitrogen. The adsorption energy for each structure is also listed below the structure. Cyan, pink, orange, gray, yellow and blue balls represent Pd, B, P, C, S and N atoms, respectively.

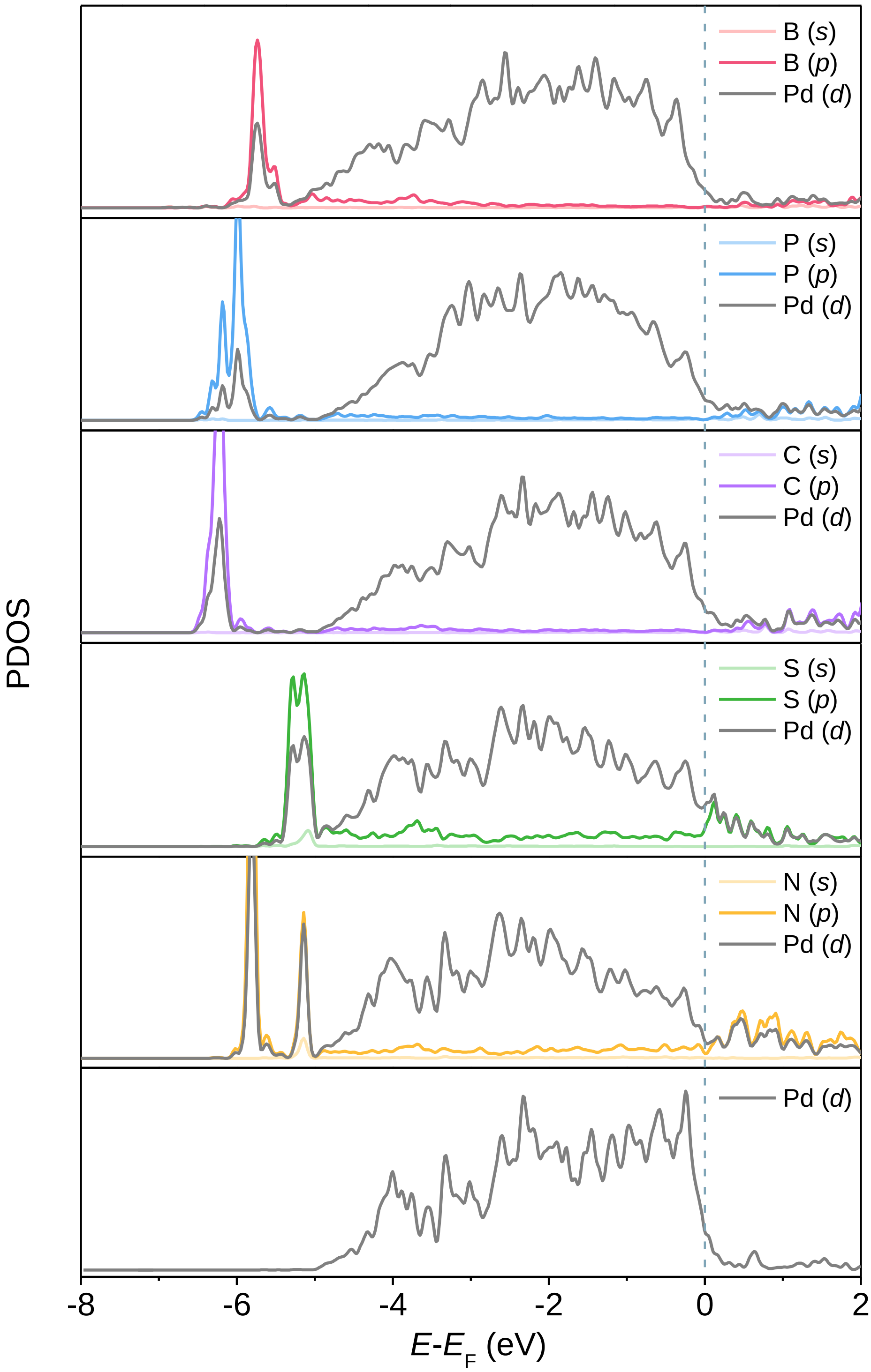

Supplementary Fig. 16. Projected density of states (PDOS). PDOS of the *d*-states of the adjacent Pd atom, *p*-states and *s*-states of non-metal interstitial atoms including B, P, C, S and N. For comparison, the pure Pd was also provided.

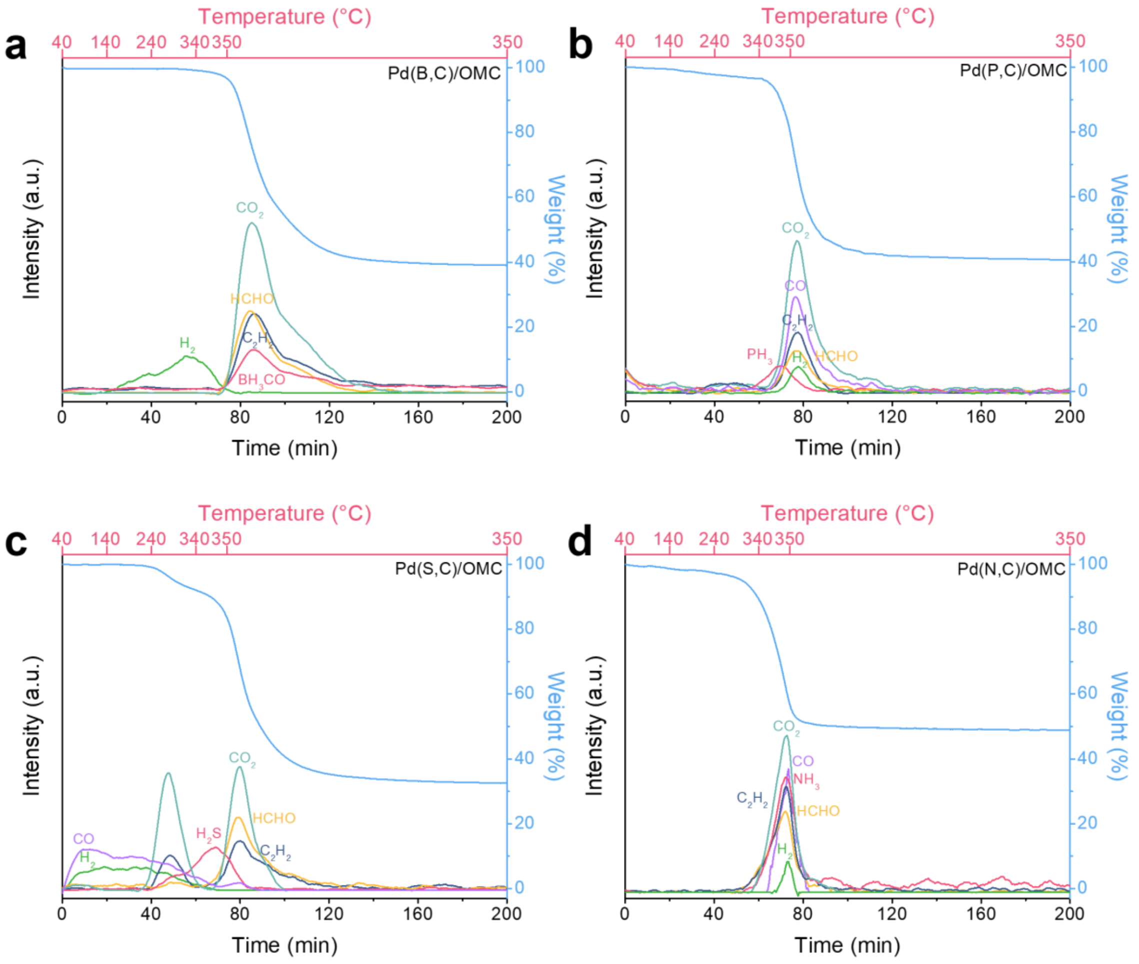

Supplementary Fig. 17. Thermogravimetry-mass spectrometry (TG-MS). Analysis of the outlet gases from the decomposition of as-made **(a)** Pd(B,C)/OMC, **(b)** Pd(P,C)/OMC, **(c)** Pd(S,C)/OMC and **(d)** Pd(N,C)/OMC by thermogravimetry-mass spectrometry (TG-MS). The TG curves revealed that approximately 38, 41, 32 and 48 wt% solid residue remained, respectively.

a

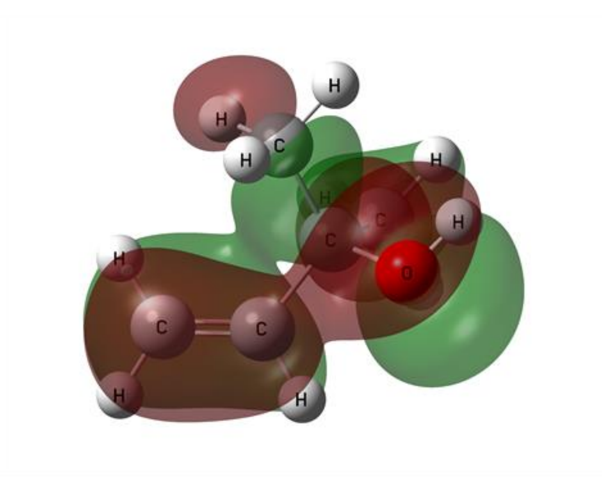

b

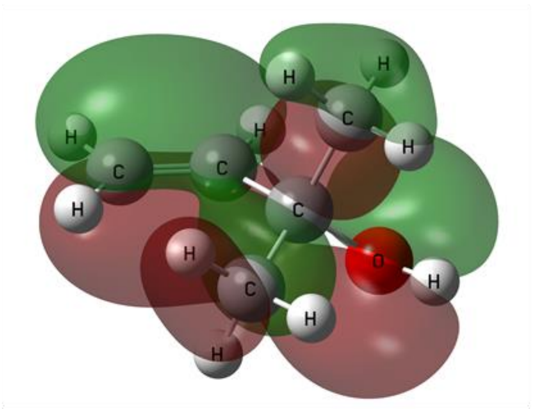

Supplementary Fig. 18. Highest occupied molecular orbital (HOMO) of MBE. (a) Top views. **(b)** Side views.

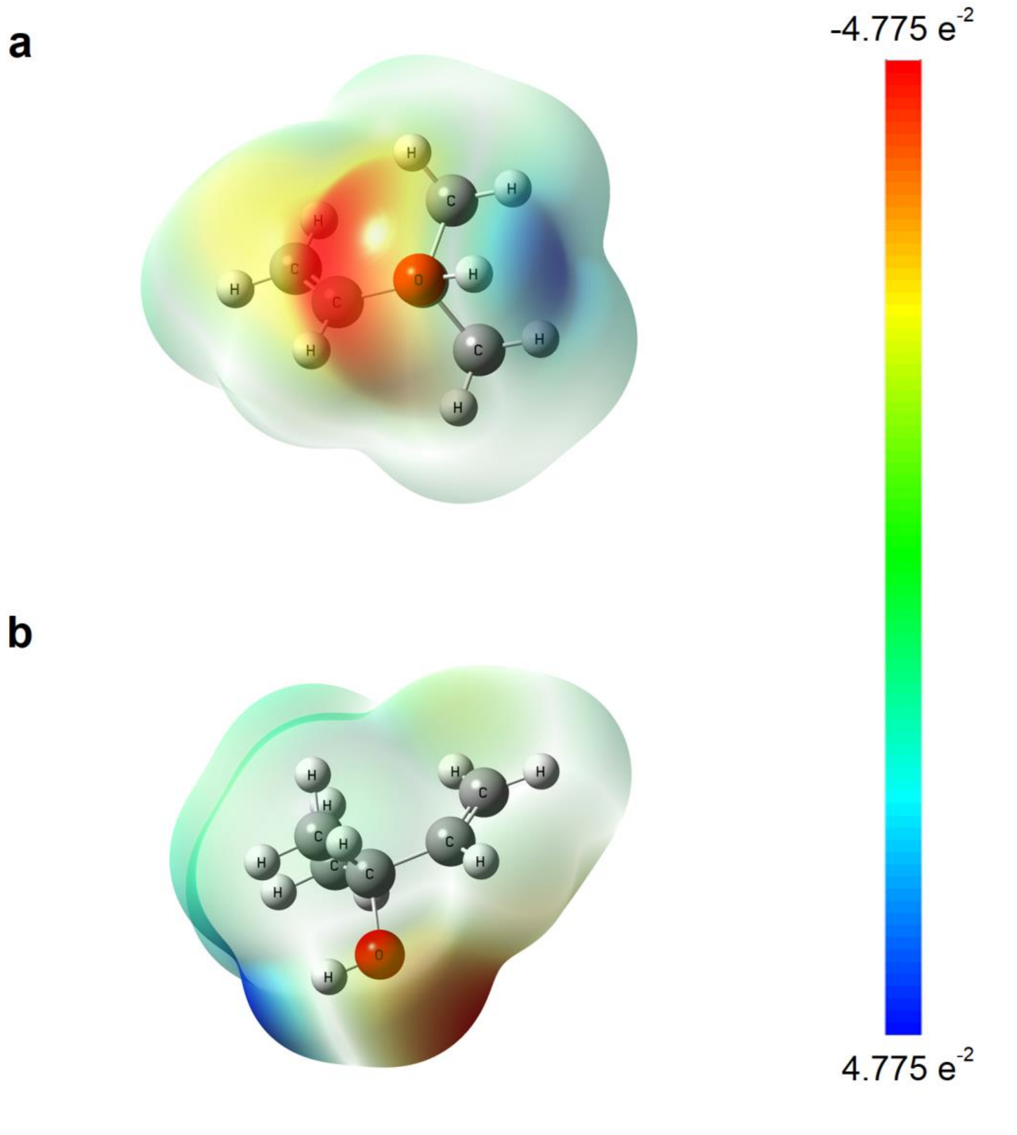

Supplementary Fig. 19. Electrostatic potential of MBE. (a) Top views. **(b)** Side views. Blue: positive, Red: negative.

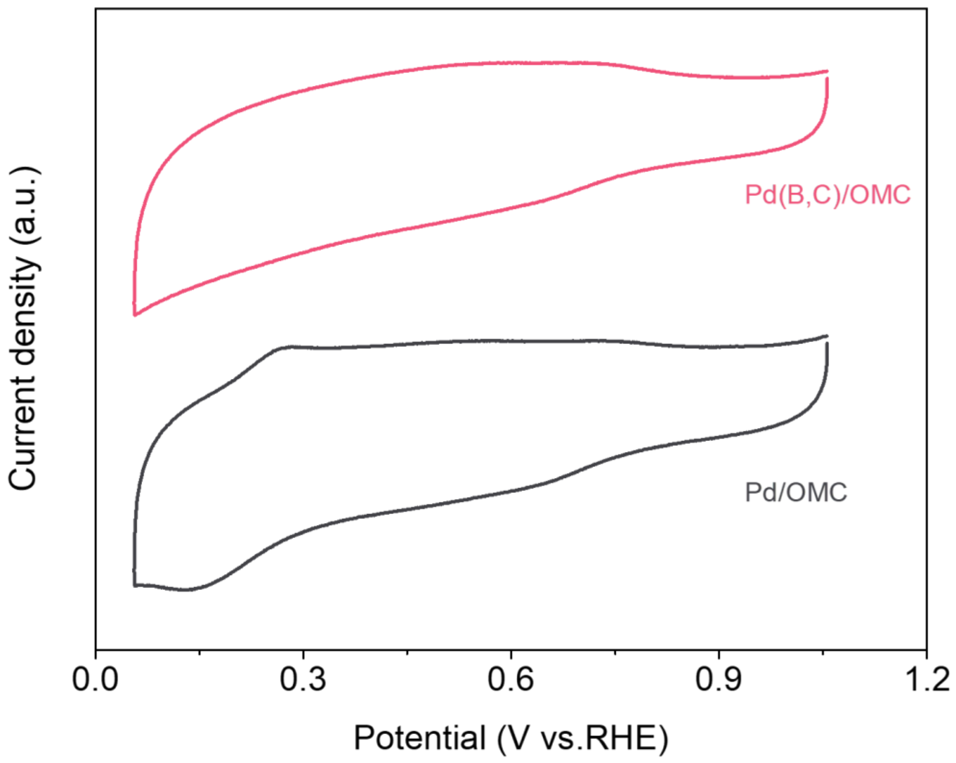

Supplementary Fig. 20. Electrochemical H adsorption experiment. Cyclic voltammograms obtained for Pd(B,C)/OMC and pure Pd/OMC in N_2-purged 0.1 M $HClO_4$ as a function of the negative potential limit.

Supplementary Table 1. The structural and textural properties of supported Pd interstitial nanocatalysts. For comparison, the properties for pure Pd/OMC and commercial Pd/C and Lindlar catalysts are also provided. Pd(B,C)/OMC-R8 is the Pd(B,C)/OMC catalyst after eight catalytic runs.

Catalyst	Pd loading[a] (wt%)	*p*-block atoms content (at%)		D_{Pd}[d] (nm)	D_{Pd}[e] (nm)	D_{Pd}[f] (nm)	S_{BET} ($m^2\ g^{-1}$)	V_p ($cm^3\ g^{-1}$)	D_P (nm)
		XPS[b]	EDX-STEM[c]						
Pd(B,C)/OMC	0.91	1.46(B)	/	5.2	5.7	5.5	503	0.54	5.2
Pd(B,C)/OMC-R8	0.90	1.41(B)	/	4.8	5.3	5.6	411	0.43	5.4
Pd(P,C)/OMC	0.93	1.32(P)	1.48(P)	4.8	5.1	4.3	578	0.57	6.1
Pd(C)/OMC	1.01	/	/	4.6	4.8	4.8	596	0.59	6.0
Pd(S,C)/OMC	1.04	1.53(S)	1.67(S)	4.1	4.3	4.1	527	0.55	5.9
Pd(N,C)/OMC	0.97	2.04(N)	2.11(N)	4.4	5.1	4.5	473	0.48	4.6
Pd/OMC	1.05	/	/	5.0	4.6	4.7	513	0.51	6.3
Pd/C catalyst	5.00	/	/	5.1	5.5	3.9	1333	0.82	1.4
Lindlar catalyst	5.00	/	/	-	5.4	4.3	-	-	-

[a]Pd loading measured by inductively coupled plasma-atomic emission spectrometry (ICP-AES);

[b]Content estimated from the XPS spectra;

[c]Content estimated from the EDX pattern collected in STEM mode;

[d]Particle size calculated by Scherrer formula;

[e]Particle size calculated by CO pulse chemisorption;

[f]Particle size estimated from the TEM images.

Supplementary Table 2. The most favourite adsorption configuration, adsorption energy and C≡C bond length of MBY on pure Pd(111) and non-metal modified Pd(111): through a di-σ configuration. For comparison, the C≡C bond length of a free MBY molecule is also provided.

Surface	Configuration	C≡C bond length (Å)	E_{ads} (eV)
Pd(111)	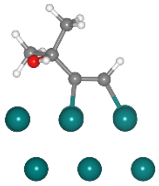	1.33	-1.18
N-Pd(111)	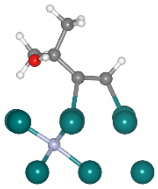	1.32	-0.95
P-Pd(111)	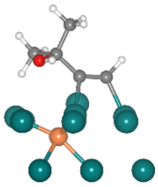	1.32	-0.95
B-Pd(111)	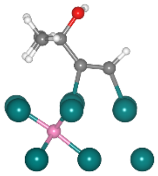	1.33	-1.09
Free MBY	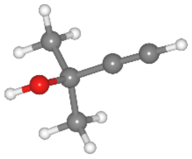	1.21	-

Supplementary Table 3. The most favorite adsorption configuration, adsorption energy and C=C bond length of MBE on pure Pd(111) and non-metal modified Pd(111): π-bonded configuration through a C=C bond on Pd(111) and N-Pd(111); a perpendicular configuration using the -OH bond on B-Pd(111) and P-Pd(111). For comparison, the C≡C bond length of a free MBY molecule is also provided.

Surface	Configuration	C=C bond length (Å)	E_{ads} (eV)
Pd(111)	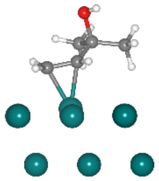	1.41	-0.37
N-Pd(111)	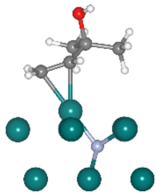	1.38	-0.68
P-Pd(111)	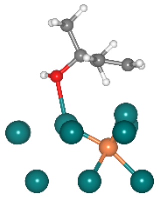	1.36	-0.51
B-Pd(111)	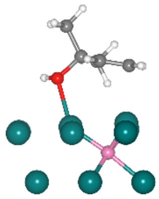	1.34	-0.43
Free MBE	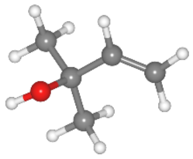	1.33	-

Supplementary Methods.

Synthesis of phenolic resins.

The carbon precursors (low molecular weight, soluble phenolic resins) were prepared from phenol and formaldehyde[1]. In a typical synthesis, 16.0 g of phenol, and 3.4 g of a 20 wt% aqueous sodium hydroxide (NaOH) solution were mixed in a round bottom flask, and heated to 45 °C. After 15 min, 28.2 g of 37 wt% formaldehyde was added. The mixture was then heated at 70 °C for 1 h with 360 rpm stirring. Next, the pH value was adjusted to ~7.0 with a 2 M HCl solution, and the superfluous water was removed by vacuum rotary evaporator below 45 °C. The soluble phenolic resins were dissolved in ethanol (20 wt%) for further use.

Synthesis of B-OMC.

B-doped ordered mesoporous carbon (B-OMC) was synthesized by a solvent evaporation induced self-assembly (EISA) method with copolymer F127 as a structure directing agent in an ethanol solution. In a typical preparation, 1.0 g of F127 and 0.5 g of triphenyl borate were dissolved in 20.0 g of ethanol. Then 5.0 g of phenolic resin in an ethanol solution containing 0.61 g of phenol and 0.39 g of formaldehyde was added. After stirring for 10 min, a homogeneous solution was obtained. The solution was poured into dishes to evaporate the ethanol at room temperature for 12 h, followed by heating in an oven at 120 °C for 24 h. The products, transparent films, were scraped from the dishes. Calcination was carried out in a tubular furnace under an inert atmosphere with a flow rate of 90 cm^3 min^{-1} at 350 °C (heating rate of 1 °C min^{-1}) for 5 h. The mesoporous products calcined were labeled B-OMC.

Synthesis of Pd/OMC.

Palladium was introduced on B-OMC by incipient wetness impregnation using palladium chloride ($PdCl_2$, >99 wt%, Shanghai Chemical Co., targeting 1.0 wt% Pd loading in the final catalyst) dissolved in ethanol acidified by hydrochloric acid as the precursor solution (Pd concentration ≈ 56.4 mmol L^{-1}). 340 μL of the palladium chloride solution was dropped onto 200 mg B-OMC, homogenized, and subsequently dried at 60 °C under vacuum for 12 h. The resulting powders were thermally reduced at 200 °C (heating rate of 1 °C min^{-1}) in a mixed gas (20 vol.% H_2/N_2) for 2 h. The obtained composite catalysts were named Pd/OMC.

Synthesis of SH-SBA-15.

SH-SBA-15 was synthesized by grafting mercaptopropyl groups on commercial mesoporous silica SBA-15[2,3]. Pristine mesoporous silica SBA-15 was synthesized by a hydrothermal method[4]. In a typical procedure, 4.1 g of SBA-15 was extracted with 120 mL of toluene in a micro-soxhlet extractor under a nitrogen atmosphere. Then, 10.0 g 3-mercaptopropyltrimethoxysilane and 1.8 mL of water were added and mixed at 110 °C for 48 h under a nitrogen atmosphere. The solids were then filtered and washed with copious amounts of toluene, ethanol, and water to remove residual silanes. The white solids were collected, dried at 150 °C overnight and stored in a vacuum dryer.

Small-angle X-ray scattering.

SAXS patterns were taken on a Bruker Nanostar U SAXS system using Cu *K*α radiation (40 mV, 35 mA).

Thermogravimetry-mass spectrometry.

The TG-MS analyses were performed on a Rigaku Thermo Mass Photo TG-DTA instrument. First, the as-made products (ca. 150 mg) were dried at 100 °C for 1 h and then cooled to 40 °C in flowing high purity Ar (>99.999%). Next, the sample were calcined in an Ar atmosphere at 350 °C, and held at 350 °C for 160 mins. The heating rate was 5 °C min^{-1}. Analysis of the outlet gases from the decomposition of as-made products a by mass spectrometer.

N_2 adsorption-desorption isotherms.

N_2 adsorption-desorption isotherms were measured at 77 K with a Micromeritics TriStar II 3020 instrument. The specific surface area (S_{BET}) of the material was obtained by the Brunauer-Emmett-Teller (BET) method. The pore volumes (*V*p) and pore size distributions (*D*p) were derived from the adsorption branches of the isotherms following the Barrett-Joyner-Halenda (BJH) model.

Inductively coupled plasma-atomic emission spectrometry.

The palladium loadings were measured with a Varian VISTA-MPX (ICP-AES) after dissolving them with a mixture of hydrochloric acid (HCl, Sinopharm Chemical Reagent Co. Ltd, 36.0-38.0 wt%) and nitric acid (HNO_3, Sinopharm Chemical Reagent Co. Ltd, 65.0-68.0 wt%) at a volume ratio of 3:1.

CO pulse chemisorption.

The dispersion of supported Pd catalyst was measured by pulsing CO adsorption on a Micromeritics Auto Chem II 2920 system. Catalysts were pretreated in 10 vol.% H_2/Ar at 100 °C for 1 h then cooled to room temperature by pure He flowing at a of 20 mL min^{-1}. Analyses were taken by pulsing research grade CO on the pretreated catalyst. Ten pulses were recorded per analysis and referenced to a blank run obtained without adding any solid catalyst. The loop volume used was 0.5 mL with pulse decay set to 15 min. A 1:1 CO to metal stoichiometric factor for dispersion calculations was used.

Electrochemical H adsorption experiment.

Electrochemical experiments were carried out in a traditional three-electrode system using an electrochemical workstation (CHI760E). A traditional three electrode system, in which a catalyst modified glassy carbon (GC) electrode, a graphite rod and a reversible hydrogen electrode (RHE) were served as working electrode, auxiliary electrode and reference electrode, respectively. The catalyst ink was prepared by mixing 5 mg of the catalyst with 250 and 15 μL of ethanol and 5 wt% Nafion (Dupont, USA), respectively, under ultrasonication for 15 minutes. 5 μL microliters of the ink was dropped onto the GC working electrode and then dried in air at room temperature. The Pd metal loading of the catalysts on the mirror-polished glassy carbon RDE (area: 0.1256 cm^2) was about 7.5 μg cm^{-2}. Cyclic voltammetry (CV) was measured in nitrogen-saturated 0.1 M $HClO_4$, scan rate 50 mV s^{-1}. The adsorption

strength of H on Pd was qualitatively determined by the oxidation charge of the underpotentially adsorbed hydrogen (H_{ads}) on the Pd surface.

Kinetics calculations.

The TOF for the all studied Pd catalyst was calculated on the basis of the estimated number of exposed palladium atoms, at a conversion below 20%.

$$\mathrm{TOF_{Pd}} = \frac{n_{\mathrm{Sub}}X}{n_{\mathrm{Pd}}t\tau} \tag{2}$$

where n_{Sub} is the molar amount of the substrate, X is the conversion, n_{Pd} is the molar amount of Pd, t is the reaction time, and τ is the exposed surface atom dispersion. τ is measured by CO pulse adsorption.

Apparent activation energies (E_a) were calculated according to the Arrhenius equation:

$$\ln k = \ln A - \frac{E_{\mathrm{a}}}{RT} \tag{3}$$

where k is the reaction rate constant, A is the apparent pre-exponential factor, R is the universal gas constant and T is the reaction temperature. Taking into account the approximate 1/2-order reaction kinetics for H_2, the reaction rate constant (k) was calculated according to the rate equation for the chemical reaction:

$$k = \frac{r_0}{c_{\mathrm{H_2}}^{\frac{1}{2}}} \tag{4}$$

where r_0 is the rate of reaction, c_{H_2} is the saturated concentration of H_2 in ethanol.

The mole fraction X_{H_2} under different pressure of H_2 was estimated from Henry's law[5]:

$$P_{\mathrm{H_2}} = K\mathrm{X}_{\mathrm{H_2}} \tag{5}$$

where P_{H_2} is the hydrogen pressure, and K is the Henry constant. At 298 K, the value of K is 513 Mpa[6].

Under same pressure of H_2, the mole fraction X_{H_2} at different temperature was estimated as follows[7]:

$$\frac{X_1}{X_2} = \left(\frac{T_1}{T_2}\right)^{2.827265} \tag{6}$$

where X_1, X_2 are the mole fraction of H_2 in ethanol at T_1 and T_2 temperature, respectively.

The entropy of activation (ΔS^{0*}) was determined as follows.

The TOF value was expressed in the Eyring form[8,9]:

$$\mathrm{TOF} = \frac{\mathrm{k}_B\mathrm{T}}{h}\exp\left(\frac{\Delta S^{0*}}{R}\right)\exp\left(\frac{\Delta H^{0*}}{RT}\right) \tag{7}$$

where k_B, h, ΔS^{0*}, and ΔH^{0*} is the Boltzmann constant, Planck constant, entropy of activation, and enthalpy of activation, respectively.

The apparent activation energy E_a was related to ΔH^{0*} by the Temkin equation:

$$E_{\mathrm{a}} = \Delta H^{0*} + \sum n_i \Delta H_i \tag{8}$$

where ΔH_i and n_i are the adsorption enthalpies and the reaction order of reactant i, respectively.

Taking into account the approximate 1/2-order reaction kinetics for MBY or MBE. The entropy change in the activation step of the chemical reaction was closely related to the thermodynamics of the rate constant, which results in the following equation:

$$\Delta S^{0*} = R \ln \left(\frac{Ah}{k_B T e^{\frac{1}{2}} \tau} \right) \quad (9)$$

Calculation of the *d* electron gain of the Pd nanocatalysts.

The difference in the number of 4*d* electrons (*d* charge) between the samples and reference Pd catalyst was evaluated from the Pd L_3-edge XANES using the following equation (12) in the literature[10]:

$$d \text{ charge} = - \frac{[(A_{sample} - A_{Pd}) \text{ Pd } L_3 + (A_{sample} - A_{Pd}) \text{ Pd } L_2]}{10.45} \quad (10)$$

where A is the peak area of the white lines at the L_3- or L_2-edge, and 10.45 is the absorption cross-section per hole in 4*d* band of each Pd atom. In order to eliminate the size effect, Pd/OMC was used as the Pd reference catalyst. The ratio of L_3 peak area to L_2 peak area was determined to be about 2.5 according to the literature for simplification[11].

Supplementary References

1. Meng, Y. et al. A family of highly ordered mesoporous polymer resin and carbon structures from organic-organic self-assembly. *Chem. Mater.* **18**, 4447-4464 (2006).
2. Crudden, C. M., Sateesh, M. & Lewis, R. Mercaptopropyl-modified mesoporous silica: A remarkable support for the preparation of a reusable, heterogeneous palladium catalyst for coupling reactions. *J. Am. Chem. Soc.* **127**, 10045-10050 (2005).
3. Duan, L. et al. An efficient reusable mesoporous solid-based Pd catalyst for selective C2 arylation of indoles in water. *ACS Catal.* **6**, 1062-1074 (2016).
4. Zhao, D. et al. Triblock copolymer syntheses of mesoporous silica with periodic 50 to 300 angstrom pores. *Science* **279**, 548-552 (1998).
5. Wainwright, M. S., Ahn, T., Trimm, D. L. & Cant, N. W. Solubility of hydrogen in alcohols and esters. *J. Chem. Eng. Data* **32**, 22-24 (1987).
6. Purwanto, Deshpande, R. M., Chaudhari, R. V. & Delmas, H. Solubility of hydrogen, carbon monoxide, and 1-octene in various solvents and solvent mixtures. *J. Chem. Eng. Data* **41**, 1414-1417 (1996).
7. Safamirzaei, M., Modarress, H. & Mohsen-Nia, M. Modeling the hydrogen solubility in methanol, ethanol, 1-propanol and 1-butanol. *Fluid Phase Equilib.* **289**, 32-39 (2010).
8. Teschner, D. et al. In situ surface coverage analysis of RuO_2-catalysed HCl oxidation reveals the entropic origin of compensation in heterogeneous catalysis. *Nat. Chem.* **4**, 739-745 (2012).
9. Chen, W. et al. Mechanistic and kinetic insights into the Pt-Ru synergy during hydrogen generation from ammonia borane over PtRu/CNT nanocatalysts. *J. Catal.* **356**, 186-196 (2017).
10. Gatla, S. et al. Influence of Sb on the structure and performance of Pd-based catalysts: An X-ray spectroscopic study. *J. Phys. Chem. C* **121**, 3854-3861 (2017).
11. Sham, T. K. *L*-edge X-ray-absorption spectra of $PdAl_3$ and $PdCl_2$: a study of charge redistribution in compounds of an element with a nearly full 4*d* shell. *Phys. Rev. B* **31**, 1903-1908 (1985).
